# HLA-DRB1 Polymorphisms Modulate the Dynamic Compatibility between the Presentation of EBNA1_400–413_ and MBP_85–99_

**DOI:** 10.1021/acsomega.6c06047

**Published:** 2026-09-17

**Authors:** Levy Bueno Alves, Silvana Giuliatti

**Affiliations:** Department of Genetics, 28133 Ribeirão Preto Medical School, University of São Paulo, USP, São Paulo 044021-001, Brazil

## Abstract

Multiple sclerosis (MS) shows a strong genetic association with HLA-DRB1 alleles and a consistent epidemiological association with Epstein–Barr virus (EBV) infection. However, it remains unclear how HLA-DRB1 polymorphisms modulate the presentation of autoantigens and viral antigens potentially involved in cross-reactivity. In this study, peptide–HLA (pHLA) complexes formed by six HLA-DRB1 alleles associated with different MS risk profiles were investigated in interaction with CLIP_103–117_, MBP_85–99_, and EBNA1_400–413_. To this end, molecular dynamics simulations were integrated with machine learning models trained using inter-residue distances at the pHLA interface as input attributes, allowing evaluation of how different peptides modulate the conformational stability of the complexes and identification of which interface regions contribute to distinguishing alleles associated with higher and lower risk. The results show that EBNA1_400–413_ does not globally reproduce the interaction mode of MBP_85–99_ within the binding groove. Compared with MBP_85–99_ and CLIP_103–117_, EBNA1_400–413_ exhibited fewer intermolecular contacts, greater solvent exposure, and less favorable binding energy. Despite this, in the higher-risk alleles DRB1*15:01 and DRB1*15:03, complexes containing EBNA1_400–413_ showed lower conformational variability and occupied low-energy regions that partially overlapped with those observed for MBP_85–99_. The machine learning models indicated distinct discriminatory patterns for the autoantigen and the viral antigen. However, in the combined MBP_85–99_ and EBNA1_400–413_ model, the most informative pairs converged on the β:70−β:72 region, including β:71, with more pronounced differences in complexes containing EBNA1_400–413_. This result suggests that β:71 contributes to interface organization in a peptide- and allele-dependent manner. In addition, the proximity between C:R402 of EBNA1_400–413_ and the region occupied by C:K93 in MBP_85–99_ suggests a local physicochemical similarity between the complexes, despite the absence of global structural equivalence between the peptides. These findings indicate that EBNA1_400–413_ does not act as an ideal structural mimic of MBP_85–99_, but may favor partially compatible conformational states in alleles associated with higher MS risk. Thus, this study proposes a mechanism of functional mimicry dependent on pHLA interface dynamics, in which HLA-DRB1 polymorphisms modulate the presentation of self and viral peptides. This model provides a molecular basis for investigating how the interaction between HLA-associated genetic risk and the immune response to EBV may contribute to autoimmune mechanisms in MS.

## Graphical abstract

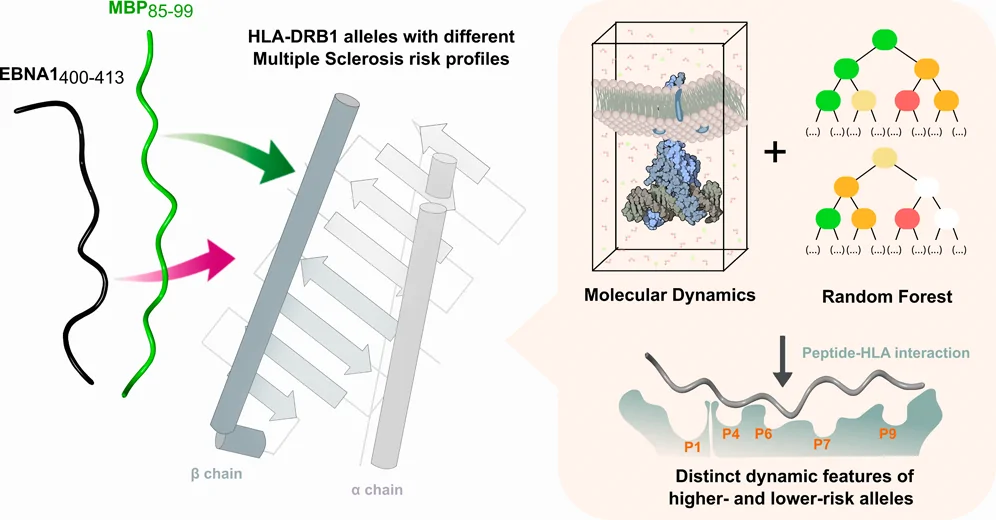

## 1 Introduction

Multiple sclerosis (MS) is a chronic neuroinflammatory disease of the central nervous system (CNS), characterized by demyelination, axonal damage, and persistent activation of adaptive immune responses against myelin components.^1^ Although the mechanisms that initiate and sustain the disease have not yet been fully elucidated, MS susceptibility is known to result from the interaction between genetic and environmental factors.^2,3^ Among genetic factors, the human leukocyte antigen (HLA) region, located on chromosome 6p21, represents the main risk factor identified for the disease, with particular emphasis on alleles of the HLA-DRB1 gene, such as HLA-DRB1*15:01 and HLA-DRB1*15:03.^4–7^ Among environmental factors, Epstein–Barr virus (EBV) infection stands out as one of the most consistent epidemiological associations with increased MS risk, suggesting that antiviral immune responses may contribute to the breakdown of self-tolerance in genetically susceptible individuals.^8–11^

The convergence between HLA-associated genetic predisposition and the immune response to viral antigens is particularly relevant, because HLA class II molecules define which peptides are presented to CD4^+^ T lymphocytes. These molecules play a central role in the adaptive immune response by presenting antigenic peptides derived from exogenous and endogenous proteins processed by antigen-presenting cells.^12,13^ From a structural standpoint, they are heterodimers composed of an α chain and a β chain. In HLA-DR molecules, the α chain is encoded by HLA-DRA and shows a low degree of polymorphism, whereas the β chain can be encoded by HLA-DRB genes. Among these genes, HLA-DRB1 exhibits a high degree of polymorphism and constitutes one of the main sources of functional variability among HLA-DR allotypes.^14,15^

In HLA-DRB1 heterodimers, the α1 and β1 domains form the peptide-binding groove. This groove accommodates peptides of variable lengths, typically between 13 and 25 amino acids, stabilized by anchor residues that preferentially interact with anchoring pockets, such as P1, P4, P6, P7, and P9.^16,17^ Because many polymorphic residues of HLA-DRB1 are located in the β1 domain, variations in this region can modify the physicochemical properties of the pockets, alter the repertoire of presented peptides, and contribute to differences in risk for autoimmune diseases.^15,18^

In MS, evidence indicates that different HLA-DRB1 alleles can modulate disease risk, with variants associated with increased or reduced susceptibility.^7,19^ Among the polymorphic sites implicated in these effects, positions β:71 and β:86 of the β1 domain stand out. The presence of alanine at β:71, shared by alleles of the DRB1*15 group, contributes to the formation of a broader and more hydrophobic P4 pocket, whereas the β:G/V86 dimorphism modulates the volume and properties of the P1 pocket.^20,21^ Thus, alleles associated with higher risk, such as DRB1*15:01 and DRB1*15:03, which carry β:A71 and β:V86, may favor binding properties distinct from those observed in structurally related alleles. This is the case for DRB1*15:02, associated with lower risk, which also carries β:A71 and is distinguished by the presence of glycine at position β:86.^7^ However, it remains unclear how these structural differences modulate the dynamics of the pHLA interface and contribute to the differential presentation of disease-associated peptides.

Among the autoantigens associated with MS, myelin basic protein (MBP) has been widely investigated because of its structural role in the myelin sheath and its ability to induce autoreactive responses mediated by CD4^+^ T lymphocytes.^20,22–26^ Experimental evidence also supports the presentation of peptides encompassing this region by HLA-DR15. In 2020, Mamedov et al. showed that MBP-derived peptides encompassing the region investigated here can be efficiently loaded onto HLA-DRB1*15:01, with MBP_85–97_ competing with an exogenous peptide for binding at concentrations starting at approximately 100 nM.^27^ On the other hand, the EBNA1_400–413_ peptide, derived from Epstein–Barr virus nuclear antigen 1, has been implicated in MS, because of its ability to stimulate immune responses similar to those induced by the autoantigen MBP_85–99_. Studies conducted by Mameli and colleagues demonstrated a pronounced humoral response against this peptide in patients with MS when compared with healthy individuals.^28,29^ In corroboration, in a fine epitope-mapping study conducted in monozygotic twins discordant for MS, Mechelli and colleagues demonstrated, in 2011, a significant increase in the humoral response against sequences located in this region, particularly the 401–411 epitope.^30^ More recently, elevated antibody responses against the broader EBNA1_381–452_ region, which encompasses EBNA1_400–413_, were detected longitudinally before MS diagnosis and were associated with subsequent disease development.^31^

One of the mechanisms proposed to connect EBV and autoimmunity in MS is molecular mimicry.^32,33^ In this model, pathogen-derived epitopes may share features with autoantigens, allowing T lymphocytes activated against the exogenous antigen to also recognize self-peptides presented by HLA molecules.^34^ In MS, cross-reactivity may occur when T cells recognize, through HLA class II, EBV-derived peptides, such as EBNA1, that mimic CNS proteins, such as MBP.^35,36^ Recent experimental evidence further supports a functional connection among EBV, HLA-DR15, and myelin autoreactivity. EBV infection in HLA-DR15-expressing humanized mice was associated with impaired viral control and the emergence of myelin-reactive T cells,^37^ while the 2026 work of Wang et al. demonstrated that EBV-infected B cells can present MBP-derived peptides through HLA-DR15 and that overlapping MBP peptides are recognized by CD4^+^ T cells from HLA-DR15^+^ patients with MS.^38^

Experimental structures of pHLA complexes provide fundamental information on how peptides are accommodated within the binding groove and on residues potentially exposed to TCR recognition. In the case of EBNA1, resolved structures are available for peptides derived from this protein in complexes with HLA class I molecules, especially the EBNA1_407–417_ epitope presented by HLA-B*35:01 and HLA-B*35:08.^39^ However, to date, no experimental structure is available for EBNA1_400–413_ complexed with HLA class II molecules. This absence limits the understanding of how this viral epitope is accommodated within the HLA-DRB1 groove and how its interface may be compared with that formed by autoantigens such as MBP_85–99_. In addition, it is important to emphasize that experimental structural models represent static conformational states and, in isolation, do not capture the dynamic variability of molecules or complexes.^40,41^ This limitation is relevant because the stability of the peptide–HLA (pHLA) interface, the persistence of molecular contacts, and the exposure of residues potentially recognized by the TCR depend on dynamic properties.

Thus, molecular dynamics (MD) simulations provide a unique perspective for investigating the conformational properties of molecular structures. In the context of pHLA complexes, this approach allows for evaluation of how different peptides influence the organization of the binding groove and the stability of the interface over time. Previous computational studies have already explored this question in HLA-DRB1 molecules belonging to the DR2 serological group related to MS. In 2013, Kumar and colleagues compared the DRB1*15:01 and DRB1*16:01 alleles in complexes with MBP_85–98_ and EBNA1_400–413_, observing that the predisposing allele DRB1*15:01 formed stable complexes with both peptides, whereas the protective allele DRB1*16:01 exhibited greater discrimination between MBP and EBNA1.^42^ However, it remains unclear how specific HLA-DRB1 polymorphisms contribute to the differential stabilization of pHLA complexes containing autoantigens and viral antigens related to MS.

In this study, we expanded this analysis to a broader set of HLA-DRB1 alleles associated with different risk profiles, aiming to evaluate whether EBNA1_400–413_ exhibits functional mimicry in relation to MBP_85–99_ and which polymorphic regions of the pHLA interface contribute to this response. To this end, we integrated MD simulations with machine learning (ML) models, since MD trajectories generate a large volume of conformational information, whose richness can be explored by ML models to identify nontrivial dynamic features. The integration of MD and ML has increasingly been explored as a strategy to extract relevant structural information from complex biomolecular simulations. In 2020, Fleetwood et al. demonstrated the potential of ML for the analysis of high-dimensional biomolecular simulation data,^43^ whereas in 2024, Weber et al. applied a combined MD–ML framework to investigate HLA-A2 immunogenicity in peptide complexes.^44^ This strategy is particularly relevant for pHLA complexes, in which risk-associated differences may be distributed across multiple inter-residue distances at the interface. Thus, ML-based models can assist in identifying residue pairs that contribute to distinguishing higher- and lower-risk alleles.

Therefore, the present study investigated whether the viral peptide EBNA1_400–413_ reproduces interaction patterns observed for MBP_85–99_ or modulates the pHLA interface through a distinct mechanism dependent on complex dynamics. The central hypothesis is that higher-risk alleles, especially DRB1*15:01 and DRB1*15:03, favor partially compatible conformational states between the autoantigen and the viral antigen and that this compatibility is influenced by polymorphic regions of HLA-DRB1 involved in the organization of the binding groove. Thus, this study seeks to contribute to the understanding of how HLA-DRB1 polymorphisms may modulate the presentation of self- and viral peptides and provide a molecular basis for mechanisms of functional mimicry associated with MS.

## 2 Materials and Methods

This section describes the procedures used for the construction, simulation, and analysis of the pHLA complexes evaluated in this study.

### 2.1 Modeling of HLA-DRB1 Heterodimers

Comparative modeling of the HLA-DRB1 heterodimer was performed using MODELLER to model regions absent from the crystallographic structure.^45,46^ The structures available in the Protein Data Bank (PDB) cover only the extracellular portion and contain structural gaps, including segments of the transmembrane domain in both subunits.^47^ In this study, the structure identified by PDB code 1YMM, determined by X-ray diffraction at 3.5 Å resolution,^48^ was used as the starting point. This structure contains the DRA*01:01 subunit (α subunit) and the DRB1*15:01 subunit (β subunit).

The first modeling step was restricted to filling the unresolved regions in the crystallographic structure, while keeping the experimentally determined atomic coordinates unchanged. For this purpose, structural models obtained from the AlphaFold Protein Structure Database were used as templates: AF–P01903-F1 for the α subunit and AF–P01911-F1 for the β subunit.^49,50^ These models were previously aligned to the crystallographic structure using Chimera, in order to preserve the same coordinate system between the templates and the experimental structure.^51^ The modeled regions were then refined in MODELLER using the loop modeling protocol, aiming to adjust their geometry to the remainder of the structure and preserve the helical conformation of the transmembrane segments.^52^ At the end of this step, a continuous model of the DRA*01:01/DRB1*15:01 heterodimer was obtained, preserving the experimental conformation of the extracellular domain.

This continuous model was then used as the reference structure to incorporate the sequence variations of the other HLA-DRB1 alleles analyzed in this study. Based on this model, structural models of heterodimers containing the DRB1*01:01, DRB1*11:01, DRB1*15:02, DRB1*15:03, and DRB1*16:01 alleles were constructed. The primary sequences corresponding to these alleles were obtained from the IPD-IMGT/HLA database under the identifiers HLA00664, HLA00751, HLA00868, HLA00870, and HLA00876, respectively.^53^ The selection of these alleles was based on their relevance to MS, as this set includes variants previously associated with different disease risk profiles.^7,19^ DRB1*15:01 and DRB1*15:03 were considered alleles associated with higher risk, whereas DRB1*01:01, DRB1*11:01, DRB1*15:02, and DRB1*16:01 were considered alleles associated with lower risk. In addition, DRB1*15:02 and DRB1*15:03 were included because they differ from DRB1*15:01 by a single point substitution at positions β:86 and β:30, respectively, allowing the evaluation of how point changes in the β-chain sequence may influence the structural and dynamic organization of HLA-DRB1 heterodimers.

### 2.2 Molecular Docking

The pHLA complexes were constructed using GalaxyPepDock, which was employed to dock the peptides into the binding groove of the previously modeled HLA-DRB1 heterodimers.^54^ For each pHLA combination, the first-ranked model returned by GalaxyPepDock was selected as the starting structure. The primary sequences of the CLIP_103–117_, MBP_85–99_, and EBNA1_400–413_ peptides were obtained from the UniProt database,^47^ under the identifiers P04233, P02686, and P03211, respectively. The MBP_85–99_ peptide was selected because it represents an autoantigen widely implicated in the pathophysiology of MS,^25^ whereas the EBV antigen EBNA1_400‑413_ was included because it stimulates immune responses similar to those induced by the autoantigen MBP_85‑99_.^29^ The CLIP_103–117_ peptide was included because it corresponds to the Class II-associated invariant chain peptide fragment, used as a structural reference because it represents the endogenous peptide naturally associated with the binding groove of MHC class II molecules during the antigen-loading process.^55^ The combination of the three peptides with the six HLA-DRB1 alleles analyzed resulted in the construction of 18 pHLA complexes.

### 2.3 Preparation of the Systems for Simulation

The resulting complexes were prepared for molecular dynamics simulations using CHARMM-GUI Membrane Builder, which enables the construction of protein–membrane systems and the generation of topology files required for the subsequent simulation steps.^56,57^ The transmembrane regions of the heterodimers were oriented along the *z*-axis using PPM 2.0, allowing the proteins to be properly positioned relative to the lipid bilayer plane.^58^ The bilayer was constructed with 800 lipids using a simplified heterogeneous composition comprising the major lipid classes found in mammalian plasma membranes.^59,60^ This composition included 45% phosphatidylcholine (DPPC), 20% phosphatidylethanolamine (POPE), 20% cholesterol (CHL1), 10% phosphatidylserine (PLPS), and 5% sphingomyelin (PSM). CHL1 was included because it is an abundant component of mammalian plasma membranes and contributes to cholesterol-dependent membrane microdomains involved in the organization of MHC-II molecules on the surface of antigen-presenting cells.^60,61^ After insertion of the heterodimers into the bilayer, each system was placed in a rectangular simulation box with approximate dimensions of 15.23 nm × 15.23 nm × 18.60 nm. The systems were then solvated with water molecules described by the TIP3P model.^62^ To approximate physiological ionic strength, the systems were electrically neutralized and supplemented with Na^+^ and Cl^–^ ions to reach a salt concentration of 0.15 M. System parameterization and topology file generation were performed using the CHARMM36m force field.^63^

### 2.4 Molecular Dynamics Simulations

Molecular dynamics simulations were performed using GROMACS v2024.4,^64^ with periodic boundary conditions applied in all three spatial dimensions. The energy-minimization and equilibration stages followed the GROMACS input protocol generated by CHARMM-GUI Membrane Builder. Before the production stage, each system was subjected to energy minimization using the steepest descent algorithm, with convergence defined as a maximum force below 1000 kJ mol^–1^ nm^–1^. The systems were subsequently equilibrated through six sequential stages, consisting of two NVT stages followed by four NPT stages. The first three equilibration stages were performed for 125 ps each, whereas the final three stages were performed for 500 ps each. During equilibration, harmonic position restraints were applied to the protein backbone and side-chain atoms, as well as to selected lipid atoms, with the corresponding restraint force constants progressively reduced over the successive stages according to the CHARMM-GUI protocol.

The equations of motion were integrated using the Verlet algorithm,^65^ with an integration time step of 1 fs during the first three equilibration stages and 2 fs during the final three equilibration stages and production simulations. A Verlet cutoff scheme was used for nonbonded interactions. Long-range electrostatic interactions were treated using the Particle Mesh Ewald (PME) method, with a cutoff radius of 12 Å,^66^ whereas van der Waals interactions were handled using a switching function between 10 and 12 Å. Bonds involving hydrogen atoms were constrained using the LINCS algorithm.^67^

During equilibration, the temperature was maintained at 303.15 K using the Berendsen thermostat, while during the NPT equilibration stages, the pressure was maintained at 1 bar using the Berendsen barostat with semi-isotropic coupling.^68^ After equilibration, production simulations were conducted for 500 ns using the Nosé–Hoover thermostat^69,70^ and the semi-isotropic Parrinello–Rahman barostat.^71^ Atomic coordinates were saved every 10 ps. All systems were simulated in duplicate. The conformational behavior of each replica was assessed using the backbone root mean square deviation (RMSD) of the HLA ectodomain, together with the bound peptide. For each system, the replica used for subsequent analyses was selected based primarily on the lowest mean RMSD, followed by the lowest standard deviation. RMSD values for both replicas are provided in the Supporting Information (Figure S1).

### 2.5 Analysis Procedures for Molecular Dynamics Trajectories

#### 2.5.1 Post-processing of Molecular Dynamics Trajectories

The trajectories were initially treated in GROMACS to correct possible artifacts arising from periodic boundary conditions.^64^ Each protein complex was then centered in the simulation box. The trajectories were subsequently superposed onto the corresponding initial structure, using the conformation at *t* = 0 ns as the reference. During superposition, global differences in rotation and translation between frames were corrected, allowing the internal conformational variations of the pHLA complex to be analyzed. After individual processing, the trajectories were aligned to a common structural reference frame using the MDAnalysis package.^72,73^ The complex containing the DRB1*15:01 allele was used as the reference, and superposition was performed based on the backbone atoms of the residues composing the HLA-DRB1 binding groove, corresponding to regions α:1–80 and β:1–93. This alignment allowed the dynamics of the binding groove to be compared across the different alleles and peptide conditions within the same spatial reference frame.

#### 2.5.2 Structural Characterization of the Lipid Bilayer

The structural organization of the bilayer was characterized by means of the area per lipid (APL), membrane thickness, acyl chain order parameter (*S*_CH_), and mass density profiles. These analyses were performed in a complementary manner to assess the structural stability of the bilayer and to evaluate the lipid packing and ordering under the temperature conditions used in the simulations.

##### 2.5.2.1 Area per Lipid

The area per lipid was calculated using LiPyphilic^74^ in conjunction with MDAnalysis 2.4.3.^72,73^ Initially, lipid molecules were assigned to the upper and lower leaflets by using the AssignLeaflets method. APL was then determined individually for each molecule using the AreaPerLipid method, which is based on a two-dimensional Voronoi tessellation in the membrane plane.^74^ For construction of the tessellation, atoms C2, C21, and C31 were used for DPPC, POPE, and PLPS; C2S, C1F, and C4S for PSM; and O3 for CHL1. Tessellations were performed separately for each leaflet, and the areas associated with representative atoms belonging to the same molecule were summed to obtain the molecular APL.

##### 2.5.2.2 Acyl-Chain Order Parameter

The *S*_CH_ order parameter was calculated from the orientation of C–H bonds relative to the bilayer normal, following the approach described for atomistic membrane simulations by Piggot et al. in 2017.^75^ The calculation was performed using a Python script based on MDAnalysis 2.4.3 ^72,73^ and NumPy, considering the coordinates and explicit connectivity between carbon and hydrogen atoms present in the topology. The acyl chains of the glycerophospholipids DPPC, POPE, and PLPS were analyzed, with the sn-1 and sn-2 chains considered separately. For each carbon, C–H vectors were determined taking periodic boundary conditions into account. When more than one hydrogen atom was bonded to the same carbon, the contributions were first averaged for that site and subsequently averaged across molecules of the same lipid species and trajectory frames. The results were reported as −*S*_CH_, separately for the sn-1 and sn-2 chains.^75^

##### 2.5.2.3 Membrane Thickness

Bilayer thickness was calculated using LiPyphilic 0.10.0,^74^ with trajectories and atom selections handled using MDAnalysis 2.4.3.^72,73^ After assigning lipids to the upper and lower leaflets with AssignLeaflets, membrane thickness was determined using the MembThickness method. Phosphorus atoms from the phosphate groups of DPPC, POPE, PLPS, and PSM were used as reference atoms, as described for the determination of phosphate-to-phosphate bilayer thickness (*D*_P–P_) in lipid bilayers.^76^ Thus, for each frame, membrane thickness corresponded to the distance along the *z*-axis between the mean positions of the phosphate groups in the upper and lower leaflets, representing *D*_P–P_. The calculation was performed using a single lateral region for the entire membrane, providing a global measure of bilayer thickness for each frame.^74^

##### 2.5.2.4 Mass Density

The distribution of membrane components along the bilayer normal was characterized using one-dimensional mass-density profiles calculated with GROMACS 2022.3.^64^ The profiles were determined along the *z*-axis, corresponding to the membrane normal, considering separately the acyl chains, cholesterol (CHL1), the glycerol/ester region, polar headgroups, and water. This analysis was used to characterize the transverse organization of different bilayer components.

#### 2.5.3 Structural and Conformational Analyses of pHLA Complexes

Trajectory analyses were performed in GROMACS, considering the peptide and the residues composing the binding groove of the HLA-DRB1 heterodimer.^64^ For the HLA chains, residues α:1–80 and β:1–93 were selected, corresponding to the α1 and β1 domains involved in the formation of the pHLA interface. The root mean square deviation (RMSD) was calculated to monitor the conformational stability of the pHLA complex throughout the simulations. The solvent-accessible surface area (SASA) was calculated for the binding-groove residues and for the peptide residues, with per-residue decomposition, allowing local differences in solvent exposure to be evaluated within the binding groove and along the peptide sequence. The secondary structure of the α1 and β1 domains was monitored throughout the trajectories using the Dictionary of Secondary Structure in Proteins (DSSP) algorithm, with the aim of verifying the conservation of the structural elements of the binding groove during the simulations. To characterize the stability of interactions between the peptide and HLA-DRB1, minimum distances over time were calculated between the atoms of the peptide and the atoms of residues α:1–80 and β:1–93. In addition, the number of intermolecular contacts between the peptide and the binding groove was quantified using a cutoff radius of 3.5 Å. Additionally, a pairwise RMSD matrix between frames was constructed, considering the backbone of the peptide and of residues α:1–80 and β:1–93. This matrix was used to evaluate the conformational variability of the pHLA complexes throughout the trajectories. Conformational clustering was performed using the GROMOS method.

#### 2.5.4 Analysis of Hydrogen Bonds at the pHLA Interface

Hydrogen bonds at the pHLA interface were analyzed throughout the MD trajectories using VMD.^77^ All frames of the simulations were considered, evaluating the interactions between the residues of the HLA-DRB1 binding groove and the peptide residues. Hydrogen bond identification was performed based on geometric criteria, adopting a maximum distance of 3.0 Å between donor and acceptor atoms and an angle cutoff of 20°. The interactions were calculated by residue pairs, allowing the frequency of occurrence of hydrogen bonds throughout the trajectories to be estimated.

#### 2.5.5 Estimation of Binding Free Energy by MM/PBSA

The binding free energy of the pHLA complexes was estimated from the last 100 ns of the MD trajectories using the gmx_MMPBSA program.^78,79^ For each system, the binding energy was calculated as the difference between the free energy of the complex and the sum of the free energies of the isolated components, defined as the HLA-DRB1 binding groove and the peptide, using an implicit solvent model. In addition to the global estimate of binding free energy, per-residue decomposition was performed to determine the individual contribution of amino acid residues at the pHLA interface to the energetic stabilization of the complex.

#### 2.5.6 Free-Energy Landscape Analysis

Free-energy landscape analysis was conducted based on the first two principal components (PC1 and PC2) obtained by Principal Component Analysis (PCA) applied to the MD trajectories after structural alignment.^80^ Calculation of the covariance matrix, extraction of the eigenvectors, and projection of the trajectories onto the principal components were performed using the GROMACS package.^64^ The coordinates projected onto PC1 and PC2 were used to map the conformational states accessible throughout the simulation. The relative free energy of each grid point was derived from the occupancy density in the PC1–PC2 space and expressed in units of kJ/mol. The output files generated by GROMACS, containing the projections onto PC1 and PC2, the free-energy values, and the occupancy probabilities in conformational space, were used as input for a Python script for data processing and analysis. Low-energy conformational states were defined as those with energies of ≤2.5 kJ/mol.^81^ The proximity between points corresponding to different peptide conditions in the same allele was evaluated using the Euclidean distance in PC1–PC2 space, with points separated by a distance of ≤0.50 units considered shared.

#### 2.5.7 Analysis of pHLA Interface Distances by Machine Learning

To identify dynamic features of the pHLA interface capable of discriminating HLA-DRB1 allele groups associated with different MS risk profiles, random forest (RF) models were constructed using distances between residue pairs as input attributes. The higher-risk class comprised DRB1*15:01 and DRB1*15:03, whereas the lower-risk class comprised DRB1*01:01, DRB1*11:01, DRB1*15:02, and DRB1*16:01.

From the MD trajectories, distances were calculated between the α-carbon (Cα) atoms of the HLA-DRB1 peptide-binding groove residues and the Cα atoms of the evaluated peptide residues. For HLA-DRB1, residues from chains α:1–80 and β:1–93 were considered. Distances were calculated in an all-to-all arrangement using the MDAnalysis v2.6.1 package,^72^ so that each trajectory frame was represented by an attribute vector corresponding to the spatial relationships between residues at the pHLA interface. Residue pairs common to all six allelic systems were retained, allowing the same set of attributes to be compared across alleles. The distances were transformed using the inverse distance (1/*d*) and each attribute was subsequently normalized to the range [0,1] using min–max scaling. Each trajectory frame constituted one instance in the machine-learning dataset, whereas the risk category assigned to the corresponding HLA-DRB1 allele was used as the class label. All frames from the 500 ns production trajectories were included. Because coordinates were recorded every 10 ps, each allele contributed 50 001 instances for each peptide condition. Three datasets were constructed for model training. The first comprised trajectories of complexes containing the autoantigen MBP_85–99_, whereas the second comprised trajectories of complexes containing the viral peptide EBNA1_400–413_. In each peptide-specific dataset, the six alleles contributed 50 001 frames each, resulting in 300 006 instances. The third dataset pooled the instances derived from the MBP_85–99_ and EBNA1_400–413_ conditions using a set of attributes common to both peptide conditions, allowing the identification of discriminatory features recurring across both systems. The combined dataset comprised 600 012 instances. The processed data were used to train RF classifiers in incremental mode with the incremental_trees library (https://pypi.org/project/incremental-trees/, accessed January 2025).^82^ Model performance was evaluated by stratified ten-fold cross-validation. Each test set contained approximately 10% of the available instances, corresponding to approximately 30 000 instances per fold for the peptide-specific models and 60 000 instances per fold for the combined model. Within each fold, the training data were shuffled and divided into blocks of 1000 instances, which were sequentially presented to the model using the partial_fit function, thereby implementing incremental training. The models were configured with n_estimators_per_chunk = 10, max_n_estimators = 1000, Gini splitting criterion, and random_state = 42. Model performance was assessed on the test set of each fold using accuracy, precision, recall, and F1-score metrics, together with confusion matrices. After cross-validation, the final models were trained using all available instances in their respective datasets while retaining the same hyperparameters. Feature relevance was assessed using class-specific importance (CSI) derived from the reduction in Gini impurity.^82^ For each split, the impurity decrease was apportioned between classes according to their proportions at the parent node and accumulated for each attribute across all trees. CSI values were first normalized within each class to sum to 1 and then rescaled to the range [0,1] using min–max normalization for ranking and presentation. This analysis allowed the identification of the residue pairs at the pHLA interface with the greatest contribution to the classification of higher- and lower-risk alleles in each peptide condition and in the combined model.

## 3 Results

This section presents the analyses of the MD trajectories of pHLA complexes formed by six HLA-DRB1 alleles associated with different MS risk profiles in interaction with the CLIP_103–117_, MBP_85–99_, and EBNA1_400–413_ peptides. The trajectories were examined at different levels of molecular organization, ranging from the structural organization of the membrane environment and conformational features of the binding groove to the local properties of the pHLA interface. Initially, the structural organization of the lipid bilayer was characterized, followed by conformational analyses of the complexes, including structural variability, conservation of secondary structure, persistence of pHLA contacts, and solvent exposure. Next, the patterns of hydrogen bonds established between the peptides and the binding groove are described, together with estimates of the binding free energy. Subsequently, the free-energy landscapes of the complexes containing the autoantigen and the viral peptide are analyzed with the aim of comparing the conformational states accessed by these systems. Finally, RF models were trained using inter-residue distances as input attributes to identify residue pairs capable of distinguishing between higher- and lower-risk alleles. The most informative pairs indicated by the models were subsequently examined through distance analysis along the trajectories.

### 3.1 Structural Characterization of the Lipid Bilayer

To evaluate bilayer organization throughout the simulations, membrane thickness, mass density profiles, and lipid-chain order parameters were analyzed jointly across the 18 systems (Figure 1). After an initial reorganization, membrane thickness stabilized at approximately 46 Å, with low dispersion among the systems throughout most of the trajectories (Figure 1A). The mass density profiles showed an approximately symmetric distribution of membrane components across the two leaflets, with acyl chains concentrated in the hydrophobic core, polar groups located at the interfacial regions, and a marked decrease in water density toward the center of the bilayer (Figure 1B). The −*S*_CH_ order parameter revealed differences according to lipid-chain composition (Figure 1C). The sn-1 and sn-2 chains of DPPC showed higher ordering, characterized by higher −*S*_CH_ values, approaching 0.4 along the more ordered portions of the acyl chains and decreasing toward the terminal carbons. For POPE and PLPS, the saturated sn-1 chains also exhibited relatively high −S_CH_ values, whereas the unsaturated sn-2 chains showed localized reductions in −*S*_CH_ at the positions corresponding to the double bonds, indicating increased local conformational disorder. The overall organization of the system, including the pHLA complex embedded in the bilayer and the aqueous environment containing ions, is shown in Figure 1D. As a complementary analysis, the area per lipid was monitored throughout the 500 ns simulations and is presented in the Supporting Information (Figure S2). After the initial reorganization period, the mean area-per-lipid values fluctuated around 47–48 Å^2^ for DPPC, 47–49 Å^2^ for POPE, 48–49 Å^2^ for PLPS, 45–47 Å^2^ for PSM, and 24–25 Å^2^ for cholesterol, with similar profiles across the systems.

**Figure 1:**
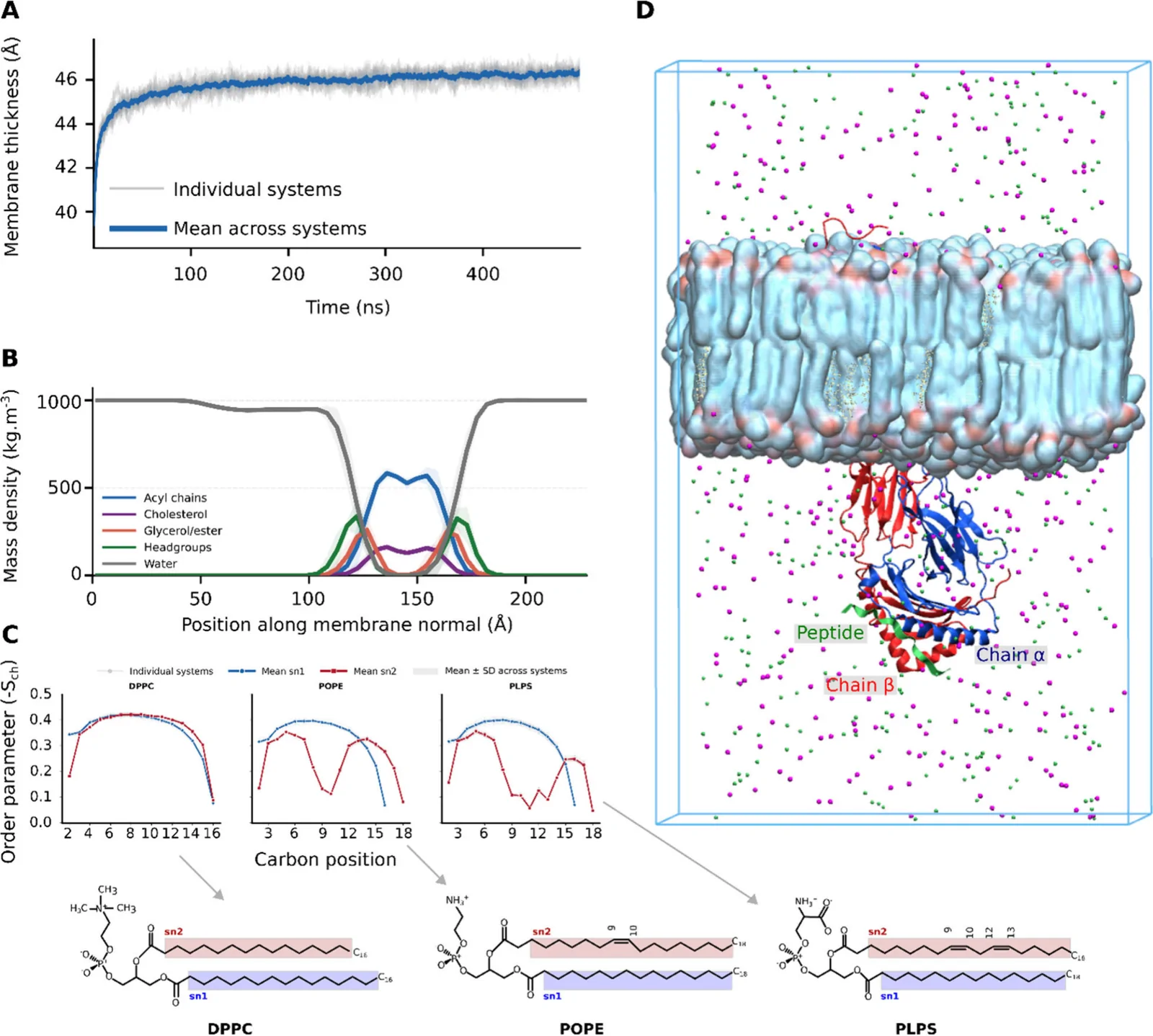
Structural characterization of the lipid bilayer in the 18 pHLA systems subjected to molecular dynamics simulations. (A) Membrane thickness over the 500 ns simulations. Gray lines represent the 18 individual systems, whereas the blue line represents the mean across systems. (B) Mean mass density profile along the axis normal to the membrane, showing the distribution of acyl chains, cholesterol, glycerol/ester regions, polar headgroups, and water. (C) Order parameter of the C–H bonds (−*S*_CH_) along the acyl chains of DPPC, POPE, and PLPS. For each lipid, mean values for the sn-1 and sn-2 chains across the 18 systems are shown together with the dispersion among systems. The chemical structures below the plots indicate the corresponding chains and the positions of unsaturations in POPE and PLPS. (D) Representation of a pHLA system embedded in the lipid bilayer and solvated in an aqueous environment containing ions, highlighting the peptide and the α and β chains of HLA-DR.

### 3.2 Conformational Analysis of pHLA Complexes

To evaluate the conformational stability of the pHLA interface, the RMSD of the backbone atoms of the residues composing the binding groove together with the peptide was calculated. As illustrated in Figure 2, all systems reached a stable state after approximately 50 ns of simulation. Comparison among peptide conditions showed that the complexes with CLIP_103–117_, MBP_85–99_, and EBNA1_400–413_ exhibited similar RMSD values, with differences lower than approximately 2 Å. However, the complex with the EBNA1_400–413_ peptide showed slightly higher mean values (3.1 ± 0.7 Å) compared with the complexes containing CLIP_103–117_ (2.5 ± 0.3 Å) and MBP_85–99_ (2.2 ± 0.4 Å). The RMSD values of the peptide-binding groove and peptide were highly similar between the simulation replicas (Supporting Information, Figure S3), indicating consistent local conformational behavior at the pHLA interface. As a complementary measure, the radius of gyration (*R*_g_) was also evaluated for both replicas and showed similar values throughout the simulations, without pronounced changes in overall compactness (Supporting Information, Figure S4). Overall, these results indicate that the peptide-binding region remained structurally stable throughout the analyzed trajectories.

**Figure 2:**
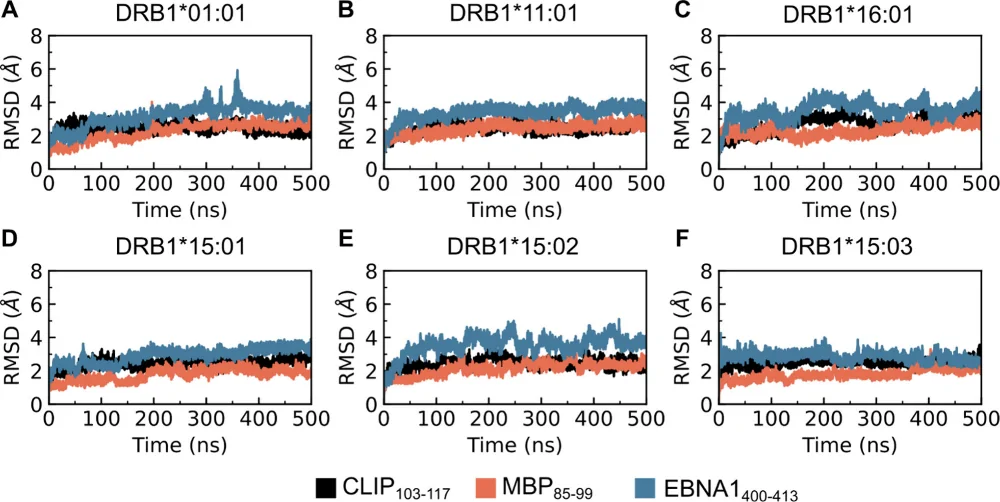
RMSD values of the pHLA complex for the HLA-DRB1 alleles under the CLIP_103–117_, MBP_85–99_, and EBNA1_400–413_ conditions. RMSD was calculated based on the backbone atoms of the binding-groove residues (chain α:1–80 and chain β:1–93) together with the peptide backbone. Results are shown for the alleles (A) DRB1*01:01, (B) DRB1*11:01, (C) DRB1*16:01, (D) DRB1*15:01, (E) DRB1*15:02, and (F) DRB1*15:03.

To characterize the conformational space sampled throughout the trajectories, an analysis based on the pairwise RMSD matrix between frames was performed (Supporting Information, Figure S5). For each system, an RMSD matrix was constructed and used to group structurally similar conformations by cluster analysis, allowing the identification of the main conformational states accessed by each pHLA complex. The distribution of RMSD values associated with the clusters reveals allele- and peptide-dependent differences in the conformational space sampled by the pHLA complexes (Figure 3). Overall, complexes with CLIP_103–117_ and MBP_85–99_ show lower dispersion of RMSD values, indicating lower conformational variability under these conditions. In the DRB1*01:01 (Figure 3A), DRB1*11:01 (Figure 3B), DRB1*15:02 (Figure 3D), and DRB1*16:01 (Figure 3F) alleles, complexes with EBNA1_400–413_ show distributions concentrated at higher RMSD values and greater dispersion, suggesting greater conformational heterogeneity of the pHLA interface. In contrast, in the DRB1*15:01 (Figure 3C) and DRB1*15:03 (Figure 3E) alleles, the distributions of MBP_85–99_ and EBNA1_400–413_ are closer and partially overlapping, indicating similar levels of conformational variability of the pHLA interface under these conditions.

**Figure 3:**
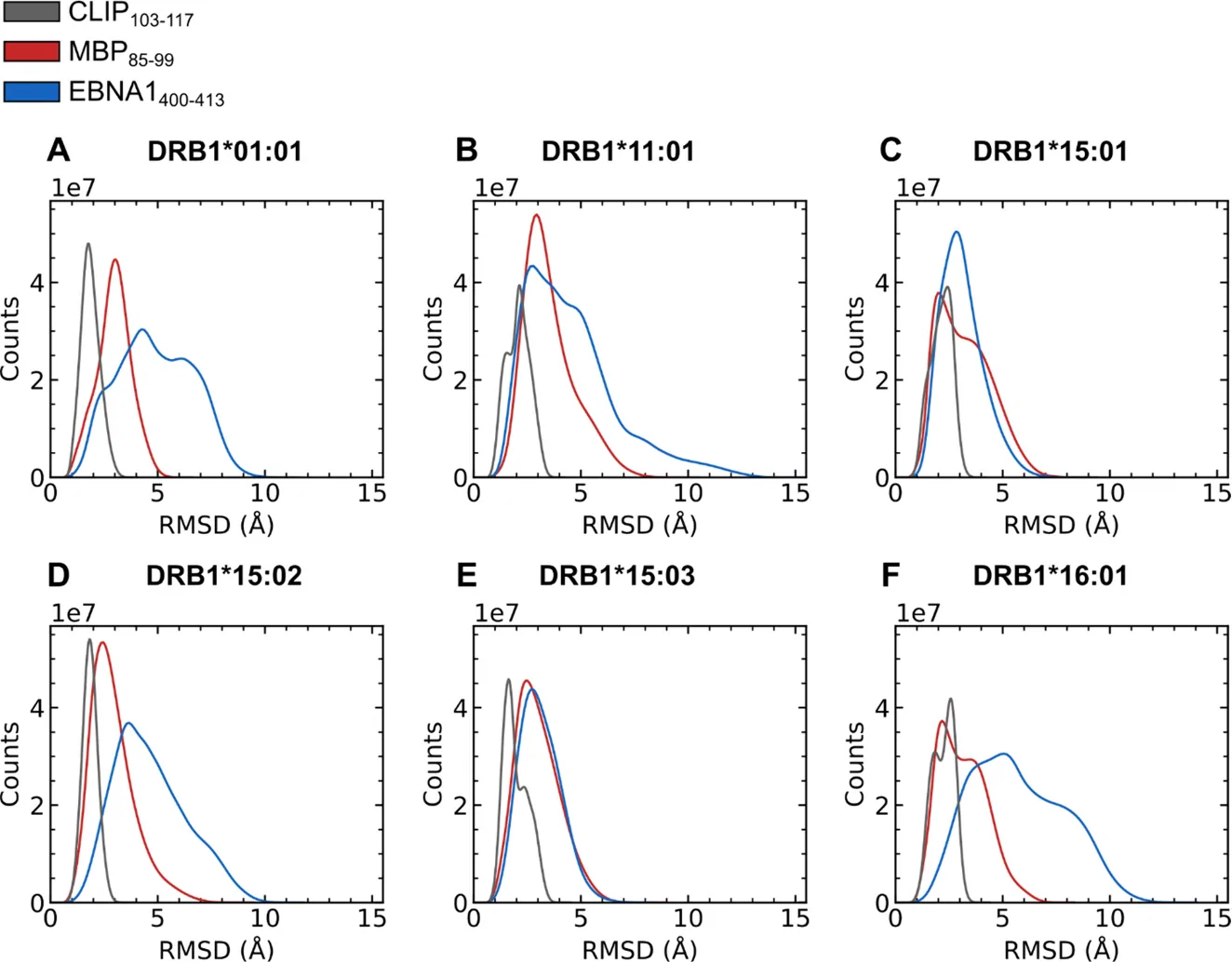
Distribution of RMSD values of the pHLA complex for different HLA-DRB1 alleles under the CLIP_103–117_, MBP_85–99_, and EBNA1_400–413_ conditions. RMSD values were obtained from the pairwise RMSD matrix between frames of the MD trajectories, considering the backbone atoms of the binding-groove residues (chain α:1–80 and chain β:1–93) together with the peptide backbone. The curves represent the frequency of occurrence (counts) of RMSD values, reflecting the conformational variability of the structural ensemble sampled during the simulations. Results are shown for the alleles (A) DRB1*01:01, (B) DRB1*11:01, (C) DRB1*15:01, (D) DRB1*15:02, (E) DRB1*15:03, and (F) DRB1*16:01.

To evaluate whether interaction with different peptides induces changes in the structural organization of the binding-groove domains, secondary structure analysis was performed using the DSSP algorithm (Figure 4). Overall, variations in the number of residues assigned to each secondary structure element in the α1 and β1 domains were conserved among the three conditions in the analyzed alleles. Only a few localized and allele-dependent differences were observed. In the α1 domain, the DRB1*01:01 allele in complex with MBP_85–99_ and EBNA1_400–413_ showed a reduction in the number of residues in α-helices, accompanied by a corresponding increase in the number of residues composing loops. Similarly, in the DRB1*11:01 and DRB1*15:02 alleles, a reduction in β-sheet content was observed in complexes with EBNA1_400–413_, with a concomitant increase in loop structures. However, in the remaining alleles, variations in α1 and β1 remained within the observed dispersion. The complete secondary structure trajectories for the different simulated alleles and conditions are presented in the Supporting Information (Figures S6–S8). These results indicate that the secondary structure of the α1 and β1 domains is globally preserved in the analyzed complexes.

**Figure 4:**
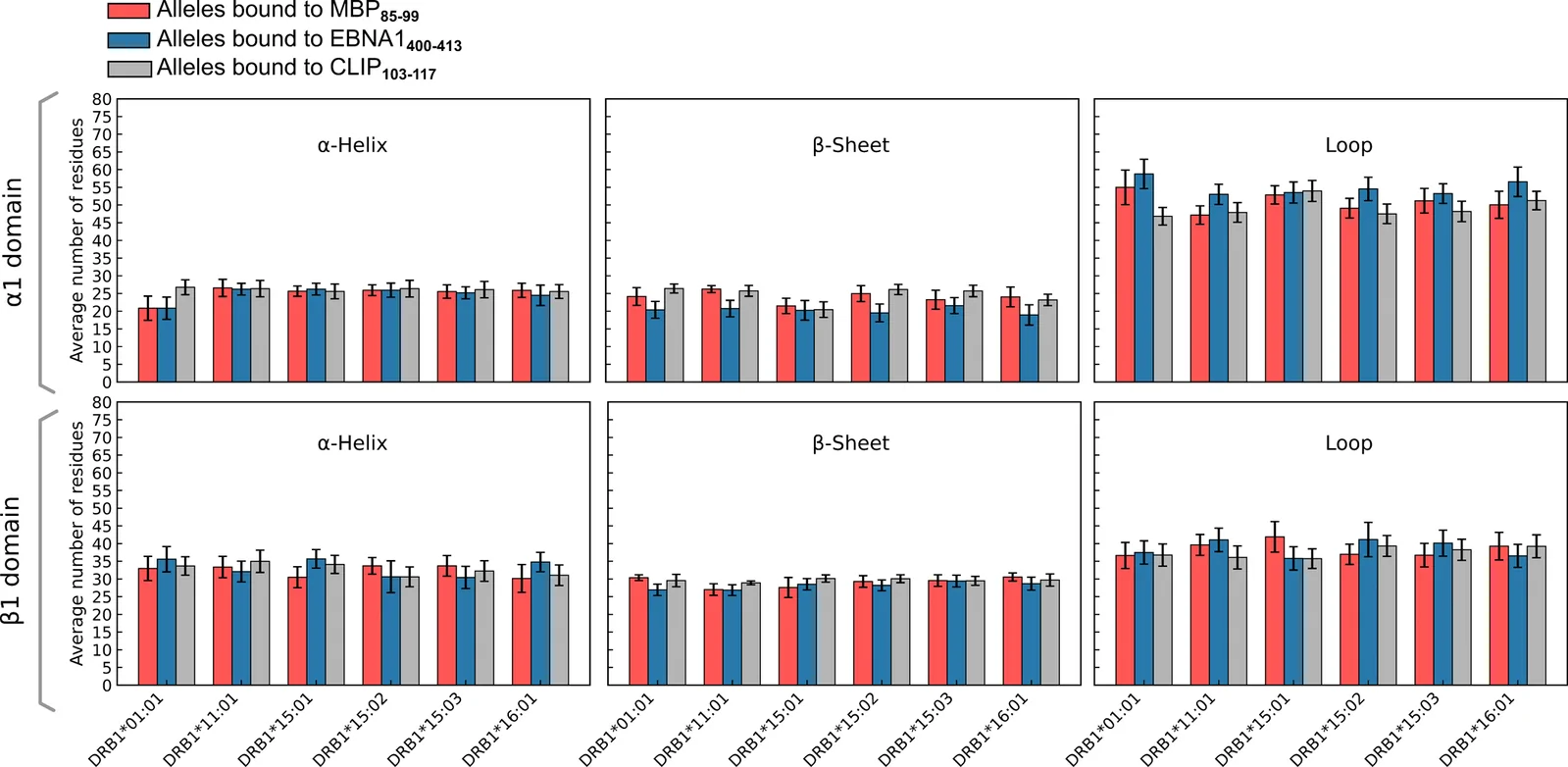
Secondary structure analysis of the α1 and β1 domains of HLA-DRB1 complexes associated with the CLIP_103–117_, MBP_85–99_, and EBNA1_400–413_ peptides throughout the MD trajectories. The plots show the mean number of residues assigned to α-helix, β-sheet, and loop for each analyzed allele. The first row corresponds to the α1 domain and the second to the β1 domain. Values represent the mean throughout the trajectory, accompanied by the standard deviation.

To evaluate the stability of interactions between the peptide and the HLA-DRB1 binding groove throughout the MD trajectories, an analysis of minimum distances between residues at the pHLA interface was performed (Figure 5). In this analysis, the time series represents, in each trajectory frame, the shortest distance observed between the atoms of a peptide residue and the atoms of an HLA residue. Thus, reduced values indicate greater proximity between the analyzed residues, whereas occasional increases reflect transient separations of these pairs during the dynamics. By definition, pHLA residue pairs are considered to interact if the distance between any atom in the peptide residue and any atom in the HLA residue is less than or equal to a cutoff distance of 4 Å.^83^ Under all evaluated conditions, the minimum distances remained below 2.4 Å over time, indicating continuous maintenance of contact between the peptide and the binding groove. As a complementary analysis to characterize the organization of the pHLA interface, the number of intermolecular contacts was quantified using a cutoff radius of 3.5 Å (Supporting Information, Figure S9). This analysis highlights differences among the peptide conditions. Complexes with CLIP_103–117_ showed, on average, the highest number of contacts (487 ± 22), followed by MBP_85–99_ (474 ± 18), whereas EBNA1_400–413_ exhibited fewer contacts (267 ± 34). These results indicate that, although all systems maintain minimum distances compatible with interaction throughout the simulation, complexes with EBNA1_400–413_ show a lower number of contacts at the pHLA interface.

**Figure 5:**
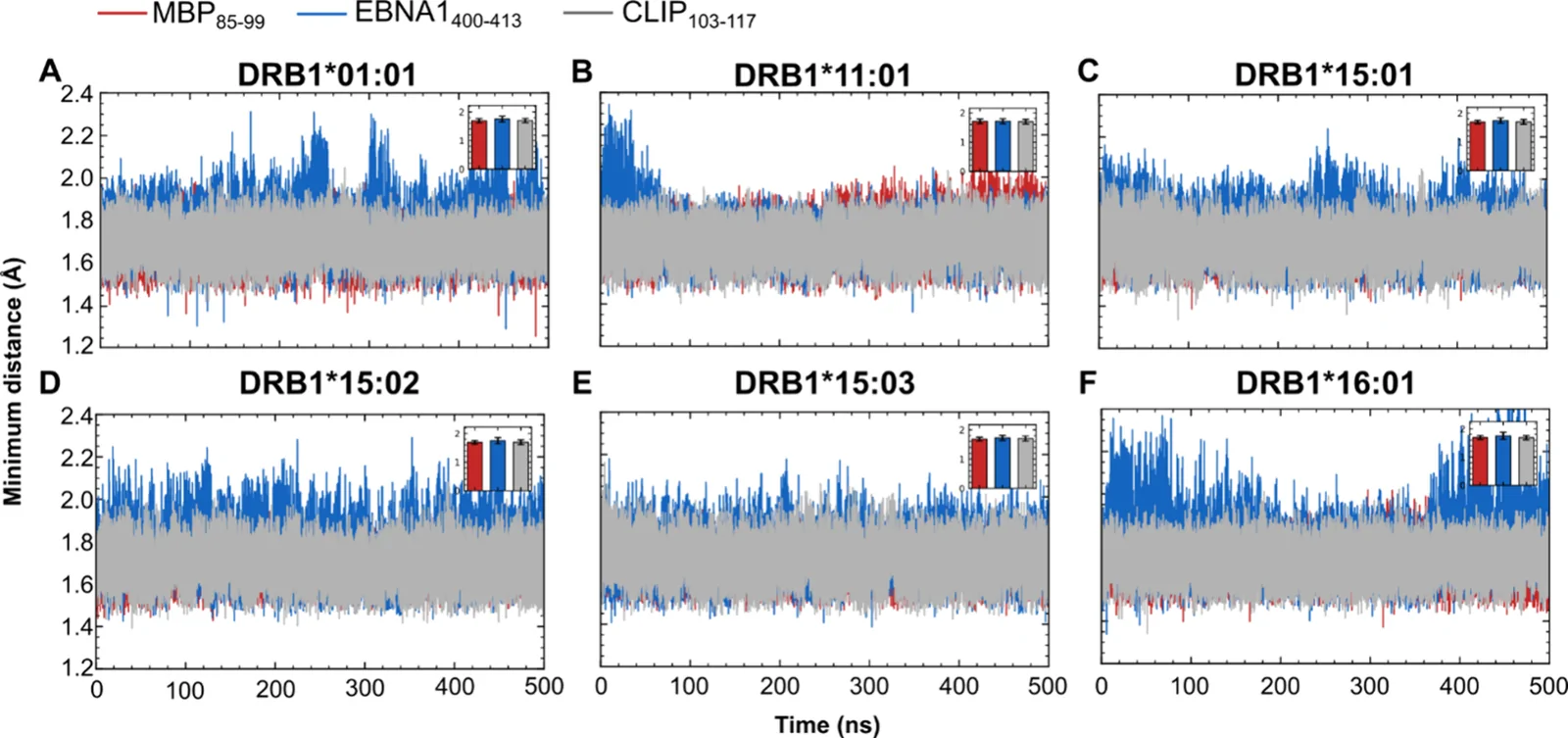
Minimum distance over time between the atoms of the peptide and the HLA-DRB1 binding groove for different alleles and peptide conditions. The curves represent the shortest distance between any peptide atom and any HLA atom throughout the MD trajectories for the CLIP_103–117_, MBP_85–99_, and EBNA1_400–413_ peptides. The panels correspond to the alleles (A) DRB1*01:01, (B) DRB1*11:01, (C) DRB1*15:01, (D) DRB1*15:02, (E) DRB1*15:03, and (F) DRB1*16:01. The inset plots show the mean minimum distance values for each condition, accompanied by the standard deviation.

The solvent-accessible surface area (SASA) was calculated for the residues composing the α1 and β1 domains of the binding groove, as well as for the CLIP_103‑117_, MBP_85‑99_, and EBNA1_400‑413_ peptides throughout the simulations. SASA corresponds to the molecular area accessible to solvent molecules and therefore allows estimation of the degree of exposure of residues or regions to the aqueous environment. Higher values indicate greater solvent exposure and vice versa.^84,85^ The values obtained for the α1 and β1 domains showed conserved behavior among the different HLA-DRB1 alleles and among the three evaluated conditions, without relevant variations along the sequence (Supporting Information, Figure S10). In contrast, differences were observed in the SASA values of the peptides (Figure 6). CLIP_103–117_ and MBP_85–99_ generally showed lower SASA values at anchoring positions in the groove, such as P1, P4, P6, P7, and P9. On the other hand, EBNA1_400–413_ showed higher values and greater SASA variation along the sequence, with more pronounced differences at positions flanking the anchoring sites, especially in the N-terminal region, where SASA values tended to remain higher than those observed for the other peptides. This behavior was recurrently observed among the analyzed alleles (Figure 6A–F), indicating that EBNA1_400–413_ generally remains more accessible to solvent than MBP_85–99_ and CLIP_103–117_, with a potentially more dynamic interaction. The highest SASA values are concentrated in specific residues of each peptide. In EBNA1_400–413_, C:R402 stands out as the main residue with high solvent exposure outside the terminal ends. In MBP_85–99_, the highest value occurs at C:K93, whereas in CLIP_103–117_ it occurs at C:R108. These residues define regions of greater solvent accessibility along the sequence and indicate a lower degree of local burial.

**Figure 6:**
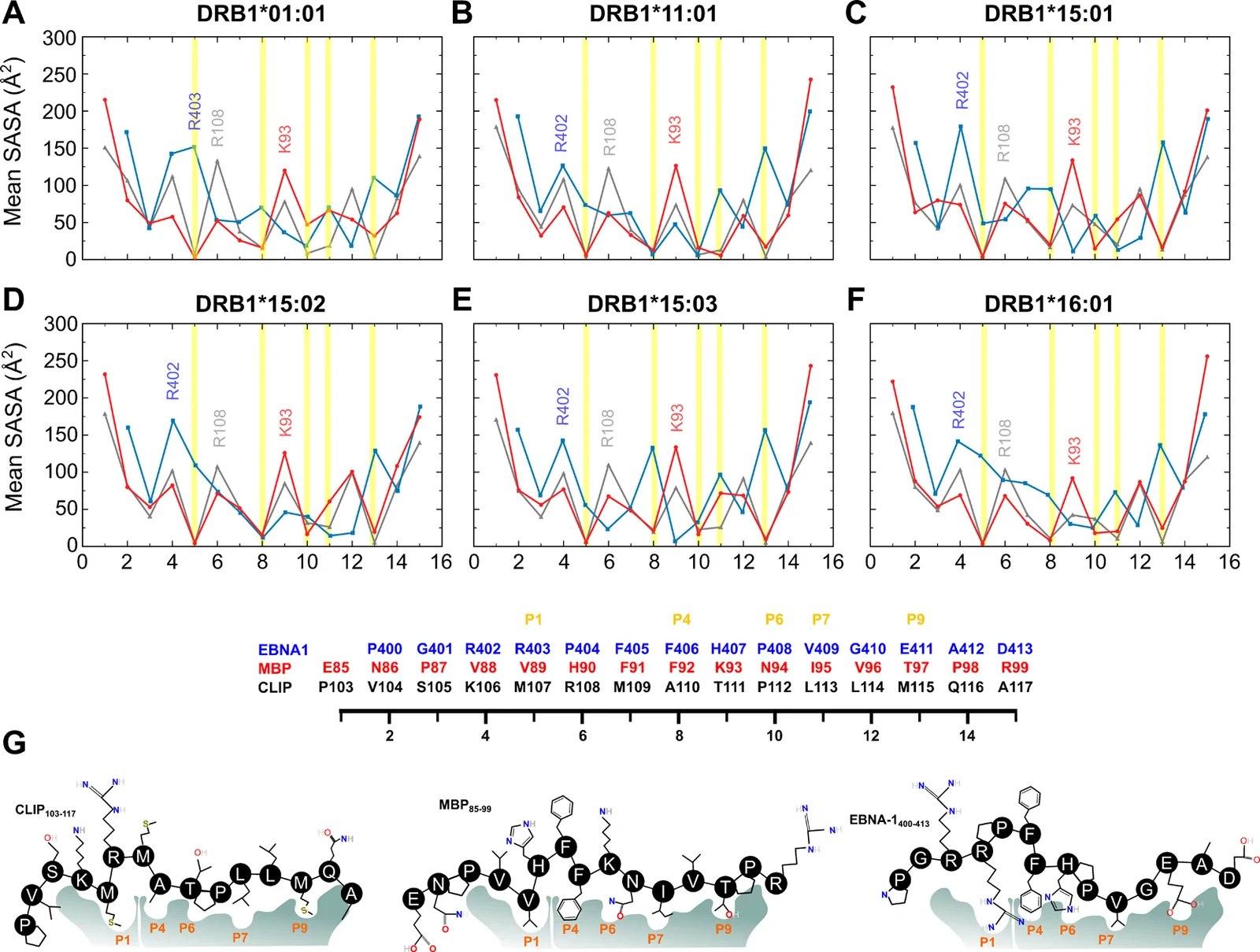
Solvent-accessible surface area (SASA) by peptide position in pHLA complexes for different HLA-DRB1 alleles. SASA was calculated throughout the MD trajectories for the CLIP_103–117_, MBP_85–99_, and EBNA1_400–413_ peptides. The panels correspond to the alleles (A) DRB1*01:01, (B) DRB1*11:01, (C) DRB1*15:01, (D) DRB1*15:02, (E) DRB1*15:03, and (F) DRB1*16:01. The regions highlighted in yellow correspond to the anchoring positions in the binding groove (P1, P4, P6, P7, and P9). The peptide sequences are indicated below the plots, allowing correspondence between the position and residue identity of each peptide. (G) Schematic representation of the CLIP_103–117_, MBP_85–99_, and EBNA1_400–413_ peptides accommodated in the HLA-DRB1 binding groove, highlighting the relative position of the residues and anchoring regions.

The results indicate that the pHLA complexes remain structurally stable throughout the trajectories with no evidence of loss of contact between the peptides and the binding groove of the evaluated HLA-DRB1 alleles. Within this conserved structural context, the differences observed among the systems are concentrated in the conformational variability of the complex and the organization of the pHLA interface. In particular, in the DRB1*15:01 and DRB1*15:03 alleles, complexes containing EBNA1_400–413_ exhibit conformational variability close to that observed for MBP_85–99_, contrasting with the behavior observed in the other alleles, in which EBNA1_400–413_ is associated with greater conformational dispersion. Additionally, EBNA1_400–413_ shows a lower number of intermolecular contacts and greater solvent exposure. These results show that, although the structural organization of the pHLA interface is preserved, peptide identity modulates the dynamics and local organization of the interface in an allele-dependent manner, with distinct behavior for EBNA1_400–413_ in the DRB1*15:01 and DRB1*15:03 alleles.

### 3.3 Analysis of Hydrogen Bonds at the pHLA Interface

To characterize the pattern of interactions that contribute to peptide stabilization within the HLA-DRB1 binding groove, hydrogen bonds at the pHLA interface were analyzed throughout the MD trajectories (Figure 7). The results show that the peptides are stabilized in the binding groove through distinct interaction networks. In complexes with MBP_85–99_, a conserved pattern is observed among the different analyzed alleles, with recurrent interactions mainly concentrated at the peptide anchor positions (P1, P4, P6, and P9). Among the most frequent interactions, C:V89−α:S53 (P1), C:H90−β:N82 (P2), C:F92−α:N62 (P4), C:N94−α:N62 (P6), and C:T97−α:N69 (P9) stand out, contributing to peptide stabilization along the binding groove (Figure 7A–F). In complexes containing CLIP_103–117_, a recurrent pattern of hydrogen bonds distributed along the groove is also observed. The most frequent interactions include C:M107−α:S53 (P1), C:R108−β:N82 (P2), and C:M115−α:N69 (P9), in addition to additional contacts such as C:Q116−β:D57 (P10) and α:N69–C:L113 (P7) (Figure 7G–L). As observed for MBP_85–99_, the interactions show occupancies below 60% and intermittent formation throughout the trajectories, suggesting that stabilization of these peptides occurs through a dynamic network of transient hydrogen bonds. In contrast, complexes containing EBNA1_400–413_ show greater variability in the hydrogen-bond pattern among the different analyzed alleles (Figure 7M–R). Unlike what was observed for MBP_85–99_ and CLIP_103–117_, the interactions are not concentrated at the same peptide positions in all complexes, being distributed across different regions of the binding groove, depending on the allele considered. This variability is reflected in the presence of persistent interactions in some alleles and more transient contacts in others. In the DRB1*15:01 and DRB1*15:03 alleles, however, recurrent interactions involving C:G410−β:D57 (P9), C:P408−β:W61 (P7), and C:R403−β:D28 (P4) are observed. Overall, the results indicate that MBP_85–99_ and CLIP_103–117_ are stabilized through a more conserved and positionally recurrent hydrogen-bond network, whereas EBNA1_400–413_ depends on more variable and allele-specific arrangements, with greater redistribution of interactions along the peptide sequence. Illustrations of the pHLA interactions are available in the Supporting Information (Figures S11 and S12).

**Figure 7:**
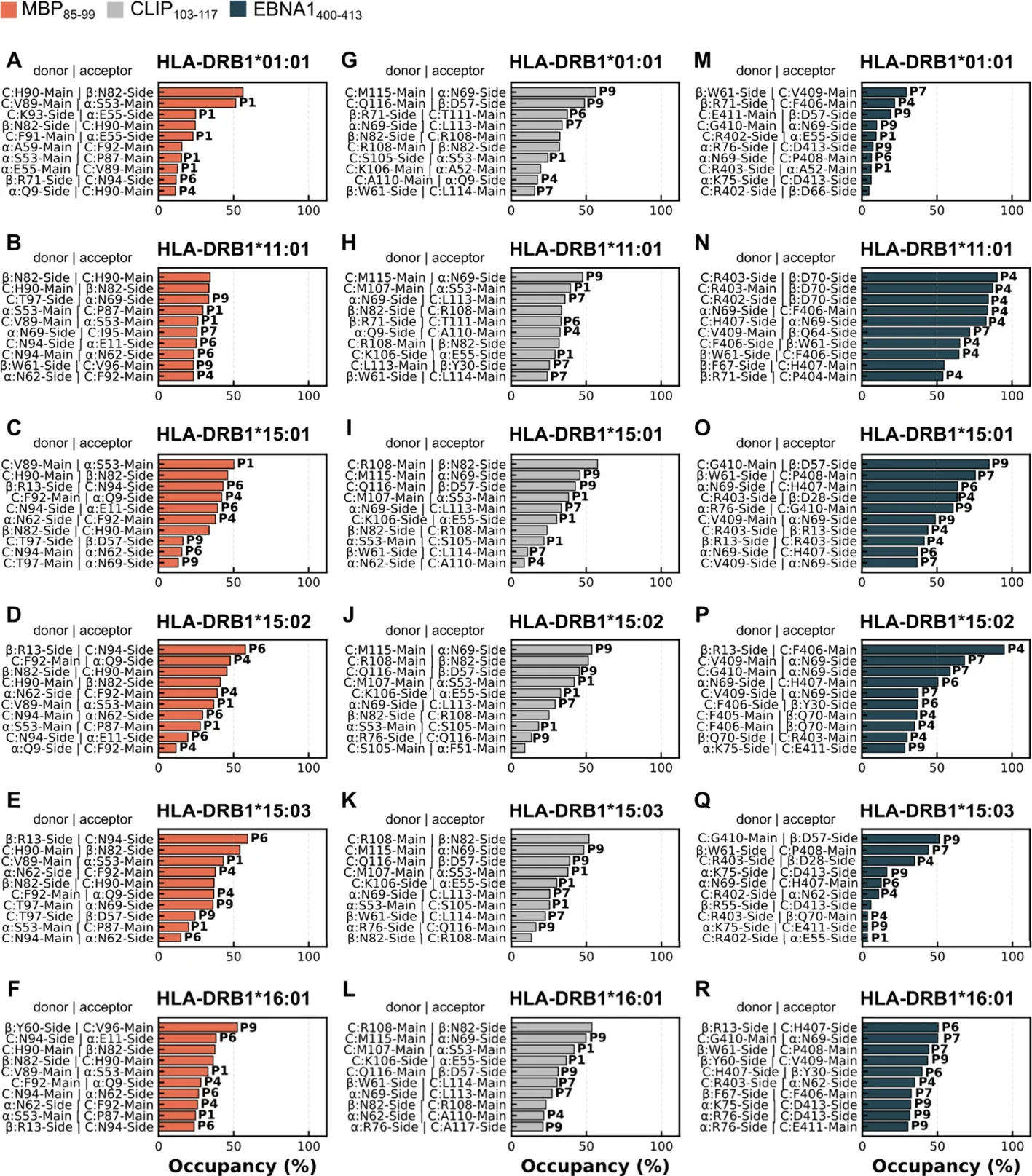
Main hydrogen bonds at the pHLA interface for HLA-DRB1 complexes associated with the MBP_85–99_ (A–F), CLIP_103–117_ (G–L), and EBNA1_400–413_ peptides throughout the MD trajectories. Each panel corresponds to a distinct HLA-DRB1 allele. The interactions are described between residues of the peptide and the binding groove chains (α and β), indicating whether they involve backbone atoms (Main) or side chains (Side). The bars represent the percentage occupancy of interactions between peptide residues and binding-groove residues (α and β chains). The residues composing the P1, P4, P6, P7, and P9 pockets were mapped as described in the 2010 work of Agudelo and Patarroyo.^86^

### 3.4 Estimation of Binding Free Energy and Per-Residue Decomposition

Binding free energy analysis was conducted using the MM/PBSA method to quantify the energetic contribution of interactions at the pHLA interface (Figure 8). The results show that complexes containing MBP_85–99_ and CLIP_103–117_ exhibit more favorable total binding free energies (ΔTOTAL) than those containing EBNA1_400–413_ (Figure 8A). For MBP_85–99_ and CLIP_103–117_, ΔTOTAL values generally fall within the range of approximately −73 to −109 kcal/mol, whereas complexes with EBNA1_400–413_ show less favorable values, between −36 kcal/mol and −48 kcal/mol. This difference is mainly attributed to the lower favorable contribution of the van der Waals (ΔVDW) and nonpolar (ΔESURF) terms for EBNA1_400–413_, reflected in less negative values for these energetic terms (Figure 8B and 8E). In contrast, electrostatic contributions (ΔEEL) do not show a uniform pattern among the alleles. In specific cases, such as DRB1*11:01, EBNA1_400‑413_ shows a more favorable electrostatic contribution, reflected in more negative ΔEEL values (Figure 8C). However, this gain is accompanied by a proportional increase in the polar solvation term (ΔEPB), resulting in compensation in the total energy (Figure 8C and 8F). In the remaining alleles, EBNA1_400‑413_ does not show a relevant electrostatic advantage (Figure 8C). The MM/PBSA results indicate that, although EBNA1_400–413_ is capable of forming stable complexes with HLA-DRB1, its energetic stabilization at the pHLA interface is lower than that observed for MBP_85–99_ and CLIP_103–117_, indicating a less favorable energetic contribution to stabilization of the pHLA interface. The distribution of energetic contributions along the pHLA interface is presented in the Supporting Information (Figure S13).

**Figure 8:**
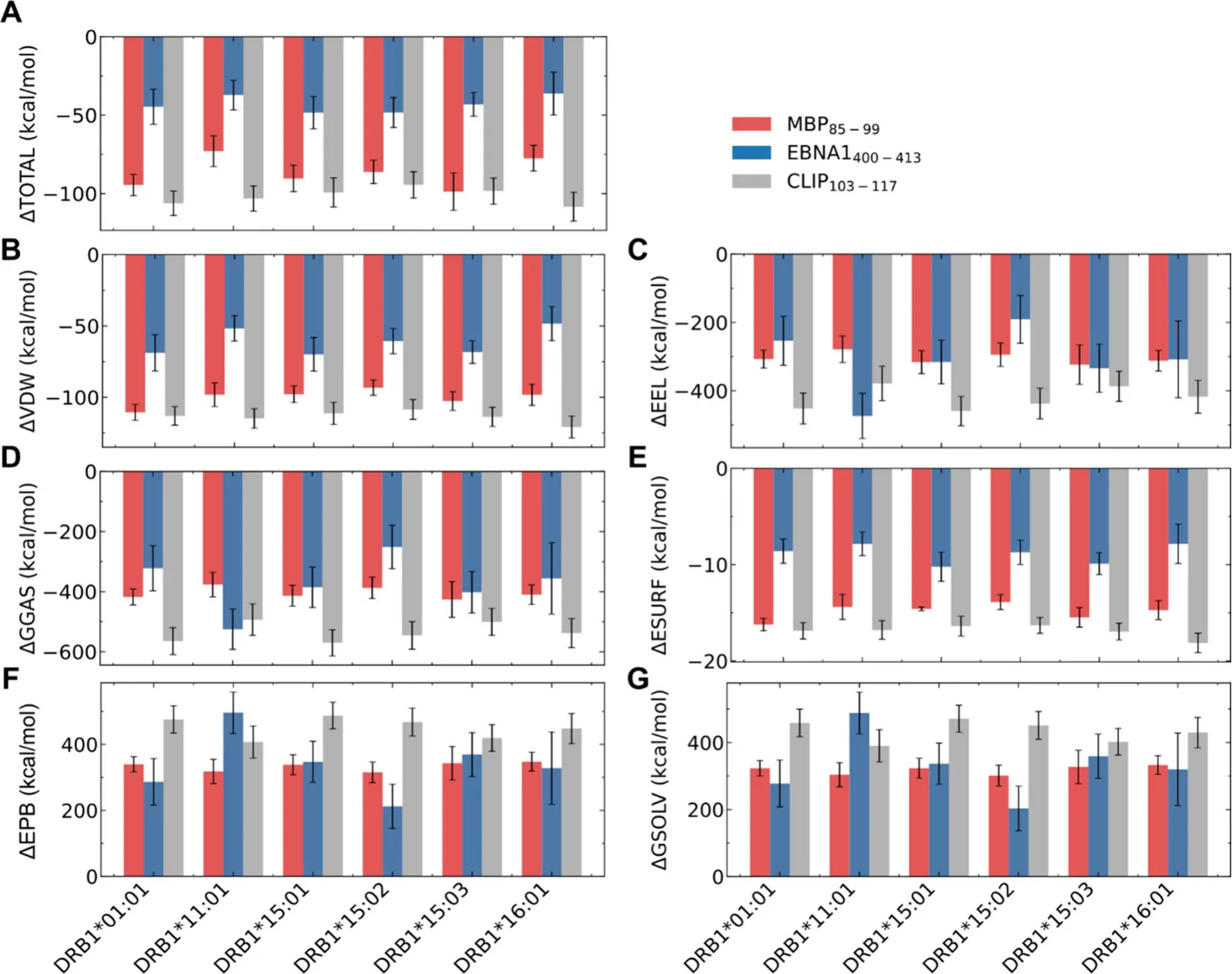
MM/PBSA binding free-energy decomposition for HLA-DRB1 complexes associated with the MBP_85–99_, EBNA1_400–413_, and CLIP_103–117_ peptides. The panels show the energetic contributions: (A) ΔTOTAL, (B) ΔVDW, (C) ΔEEL, (D) ΔGGAS, (E) ΔESURF, (F) ΔEPB, and (G) ΔGSOLV, for the DRB1*01:01, DRB1*11:01, DRB1*15:01, DRB1*15:02, DRB1*15:03, and DRB1*16:01 alleles. Values correspond to the means calculated from the last 100 ns of the MD trajectories, accompanied by the respective standard deviation.

### 3.5 Free Energy Landscape of pHLA Complexes

To evaluate how HLA-DRB1 complexes associated with the MBP_85–99_ and EBNA1_400–413_ peptides distribute their conformational states across the conformational space sampled in the MD trajectories, free-energy landscape analysis was performed. All analyses were conducted by considering the peptide together with the binding-groove residues, so that the described conformational states reflect properties of the interface potentially recognized by the TCR.^87^ In these landscapes, low-energy regions (≤2.5 kJ/mol) correspond to the most populated states throughout the dynamics. The FELs of all systems are presented in the Supporting Information (Figure S14) and show delimited low-energy regions compatible with the formation of stable or metastable states. Figure 9 presents the analysis of the DRB1*15:01 (Figure 9A) and DRB1*15:03 (Figure 9B) alleles, in which the overlap between the energy minima of the MBP_85–99_ and EBNA1_400–413_ complexes was most evident. In these alleles, the low-energy regions associated with the two peptides occupy partially coincident areas in the PC1–PC2 space, indicating that the most populated conformational states are distributed in similar regions of the conformational landscape. Quantitative data for all systems are presented in the Supporting Information (Table S1). This conformational convergence is illustrated in Figure 9C, which presents representative structures from shared low-energy regions of the HLA-DRB1*15:01 complex, the main MS risk allele. In these conformations, although local differences between the peptides can be observed, particularly in the solvent-facing N-terminal region of EBNA1_400–413_, both induce a similar organization of the HLA-DRB1 binding groove, which may be associated with the lower conformational variability observed for these systems. The most exposed residues correspond to C:R402 in EBNA1_400–413_ and C:K93 in MBP_85–99_. The binding modes of these peptides are presented in Figure 9D and 9E.

**Figure 9:**
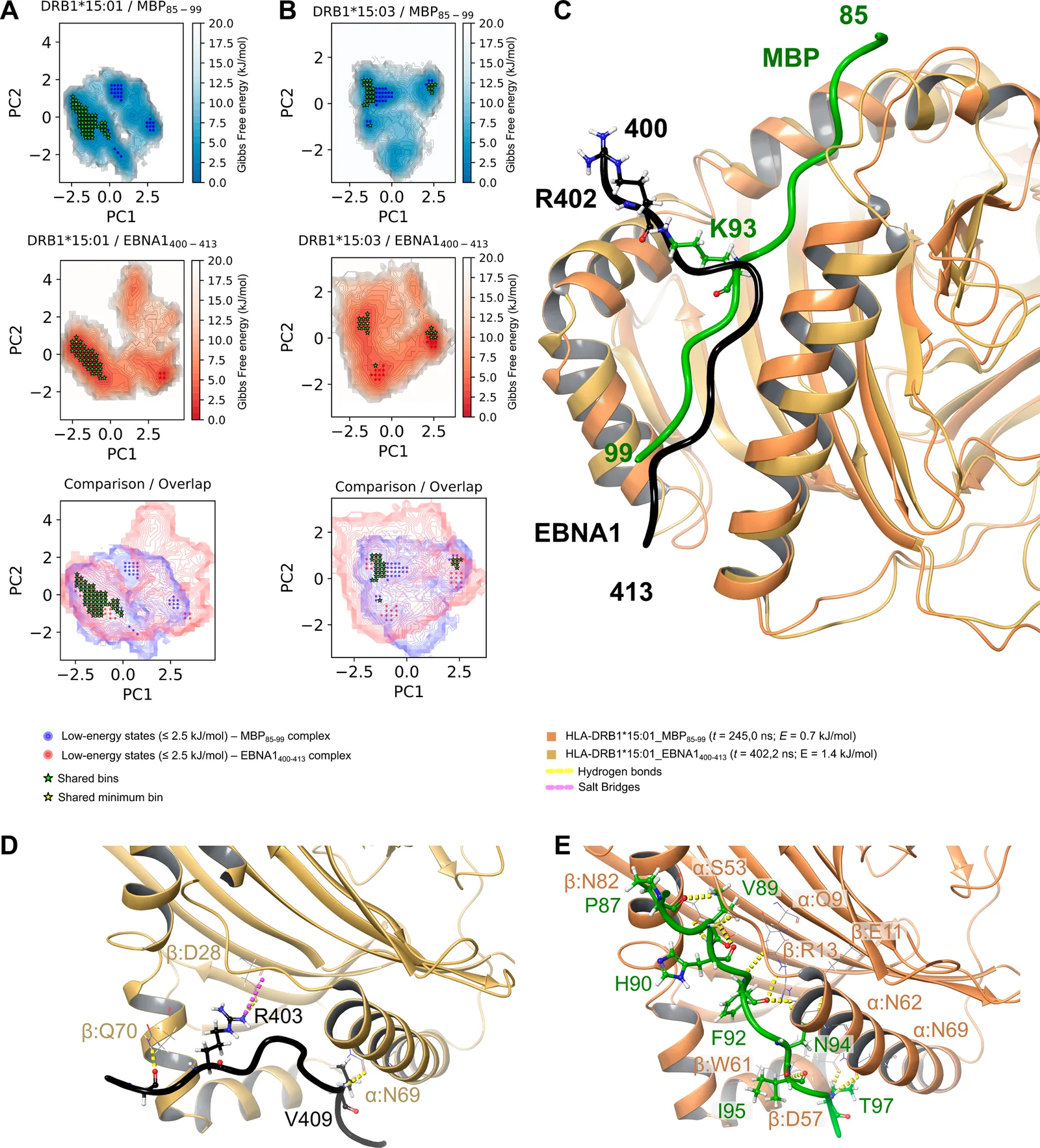
Free-energy landscapes (FELs) of pHLA complexes projected onto the principal components PC1 and PC2 for the HLA-DRB1*15:01 and HLA-DRB1*15:03 alleles under the MBP_85–99_ and EBNA1_400–413_ conditions. For each allele, the individual free energy maps for MBP_85–99_ (upper row) and EBNA1_400–413_ (middle row) are shown, as well as the overlap of low-energy regions (≤ 2.5 kJ/mol) in the PC1–PC2 plane (lower row). The FELs were calculated considering the backbone atoms of the binding-groove residues and the peptide. The colors represent Gibbs free energy (0–20 kJ/mol), with darker regions indicating more stable states. The panels correspond to the alleles (A) DRB1*15:01 and (B) DRB1*15:03. Panel (C) presents representative shared low-energy structures of the HLA-DRB1*15:01 complex, highlighting the MBP_85–99_ (green) and EBNA1_400–413_ (black) peptides accommodated in the binding groove. EBNA1-C:R402 and MBP-C:K93, previously identified in the SASA analysis as residues with high solvent exposure, are highlighted. (D) Representation of the HLA-DRB1*15:01-EBNA1_400–413_ complex. (E) Representation of the HLA-DRB1*15:01-MBP_85–99_ complex.

### 3.6 Identification of Discriminatory Residue Pairs at the pHLA Interface

To identify dynamic features at the pHLA interface capable of distinguishing HLA-DRB1 alleles associated with different MS risk profiles, ML models were trained by using distances between residue pairs at the pHLA interface as attributes. Within the studied panel of six alleles, the classification task consisted of discriminating alleles associated with higher disease risk, DRB1*15:01 and DRB1*15:03, from alleles associated with lower risk. To this end, three models were constructed: one based exclusively on complexes containing the autoantigen MBP_85–99_; another based on complexes containing the viral antigen EBNA1_400–413_; and a combined model, using attributes derived from both sets of complexes. This strategy allowed evaluation of which interactions at the pHLA interface contribute to distinguishing high- and low-risk alleles under each peptide condition within the analyzed allele panel as well as investigation of whether there is convergence of discriminatory features between the two peptides. Model performance was evaluated by stratified ten-fold cross-validation, and, overall, all models showed high performance, with an accuracy above 0.95, a macro F1-score above 0.94, and precision and recall values close to or above 0.94 (see Table S2 in the Supporting Information). Combined confusion matrices and cross-validation metrics for each model are presented in the Supporting Information (Figure S15). After validation, each RF model was trained using the complete respective datasets to obtain the final models used for feature-importance analysis (Supporting Information, Table S3). Feature importance was evaluated using CSI derived from Gini impurity reduction, allowing for identification of the ten residue pairs with the greatest contribution to classification of the high-risk class (Table 1). The ten pairs with the greatest contribution to the low-risk class are shown in the Supporting Information (Table S4).

**Table 1:** Residue Pairs at the pHLA Interface with the Greatest Contribution to the Classification of HLA-DRB1 Alleles Associated with Higher MS Risk, Identified by the ML Models^a^

	MBP_85–99_			EBNA1_400–413_			MBP_85–99_ and EBNA1_400–413_		
Rank	Pair	CSI (× 10^–3^)	Norm.	Pair	CSI (× 10^–3^)	Norm.	Pair	CSI (× 10^–3^)	Norm.
1	**α:28–C:V88**	**8.722**	**1.00**	**β:57–C:P404**	**21.817**	**1.00**	**β:71–MBP-C:F91/EBNA1-C:F406**	**17.116**	**1.00**
2	**α:26–C:V89**	**8.545**	**0.98**	**β:54–C:P404**	**18.057**	**0.83**	**β:72–MBP-C:F91/EBNA1-C:F406**	**15.066**	**0.88**
3	**α:27–C:P87**	**8.108**	**0.93**	β:57–C:H407	16.196	0.74	**β:70–MBP-C:F91/EBNA1-C:F406**	**14.119**	**0.82**
4	**α:27–C:E85**	**7.990**	**0.92**	β:55–C:H407	15.565	0.71	β:64–MBP-C:N94/EBNA1-C:V409	13.522	0.79
5	**α:26–C:E85**	**7.294**	**0.84**	β:52–C:P404	15.314	0.70	β:65–MBP-C:K93/EBNA1-C:P408	10.770	0.63
6	**α:26–C:V88**	**7.292**	**0.84**	β:29–C:R403	14.083	0.65	β:65–MBP-C:N94/EBNA1-C:V409	9.915	0.58
7	**α:26–C:P87**	**7.135**	**0.82**	β:72–C:P408	12.737	0.58	β:68–MBP-C:K93/EBNA1-C:P408	9.656	0.56
8	**β:64–C:V96**	**6.987**	**0.80**	β:61–C:P404	12.341	0.57	β:67–MBP-C:K93/EBNA1-C:P408	8.820	0.52
9	**α:27–C:N86**	**6.947**	**0.80**	β:73–C:P408	12.256	0.56	β:63–MBP-C:V96/EBNA1-C:E411	7.621	0.45
10	α:26–C:N86	6.661	0.76	β:56–C:H407	12.235	0.56	β:66–MBP-C:K93/EBNA1-C:P408	7.131	0.42

a The ten most informative pairs are shown for the models trained with complexes containing MBP_85–99_, EBNA1_400–413_, and the combined MBP_85–99_ and EBNA1_400–413_ model. Each pair is represented as a spatial relationship between a residue of the α or β chain of HLA-DRB1 and a peptide residue (chain C). For each pair, the class-specific importance (CSI, ×10^–3^) and the corresponding min–max normalized value (Norm.) are reported. In the combined model, equivalent HLA positions are shown together with the corresponding residues of the two peptides. Pairs highlighted in bold correspond to those with normalized importance values greater than or equal to 0.8.

Overall, the most informative residue pairs differed between the models based on each peptide, indicating that different regions of the pHLA interface contribute to discrimination between risk alleles, depending on the presented ligand. In the model based on MBP_85–99_, the most relevant pairs mostly involved the α chain, especially positions α:26–28, associated with residues located in the N-terminal region of the peptide, such as C:E85, C:P87, C:V88, and C:V89. In contrast, in the model based on EBNA1_400–413_, the most informative pairs mainly involved the β chain, with emphasis on positions β:57 and β:54, associated with residue C:P404. In the combined MBP_85–99_ and EBNA1_400–413_ model, the most informative pairs were concentrated in a specific region of the β chain that includes the polymorphic site β:71, previously associated with MS susceptibility.^7^ At this position, alleles of the DRB1*15 group (DRB1*15:01, DRB1*15:02, and DRB1*15:03) carry alanine, whereas DRB1*01:01, DRB1*11:01, and DRB1*16:01 carry arginine. Recurrence of pairs involving corresponding positions in the two peptides was observed, particularly MBP-C:F91 and EBNA1-C:F406, which appear associated with β:71, β:72, and β:70.

### 3.7 Analysis of the Distances of Pairs Identified by Random Forest

To evaluate whether the pairs selected by the ML models correspond to differences in the spatial relationships between residues at the pHLA interface, the mean distances of the most informative pairs in each model were analyzed (Figure 10). In the model based on EBNA1_400–413_, the discriminatory pairs predominantly involved the β chain of HLA-DRB1, with emphasis on β:57–C:P404 and β:54–C:P404 (Figure 10A). These pairs showed more pronounced differences between high- and low-risk alleles, characterized by shorter distances in the high-risk alleles. In the model based on MBP_85–99_, discriminatory pairs were also identified, but they were mainly concentrated in the α chain, especially at positions α:26–28, located in the P1 pocket. These positions are adjacent to residue β:86, which shows G/V dimorphism among the analyzed alleles, with valine present in the risk alleles (DRB1*15:01 and DRB1*15:03) and glycine in the others. In these cases, the distance differences between classes were significant but of lower magnitude, consistent with a stabilized interface with anchor residues accommodated in a conserved manner within the binding groove (Figure 10B). In the combined MBP_85–99_ and EBNA1_400–413_ model, the most informative pairs converged on the β:70−β:72 region, including the polymorphic site β:71. This position appeared associated with corresponding residues in the two peptides, mainly MBP-C:F91 and EBNA1-C:F406. Therefore, the distance analysis indicates that the effect of this region depends on the ligand. Differences involving β:71 were more pronounced in complexes with EBNA1_400–413_, whereas in complexes with MBP_85–99_, they remained more subtle (Figures S16 and S17 in the Supporting Information). Thus, β:71 constitutes a common informative region in the combined model, but with a more evident structural contribution in complexes with EBNA1_400–413_.

**Figure 10:**
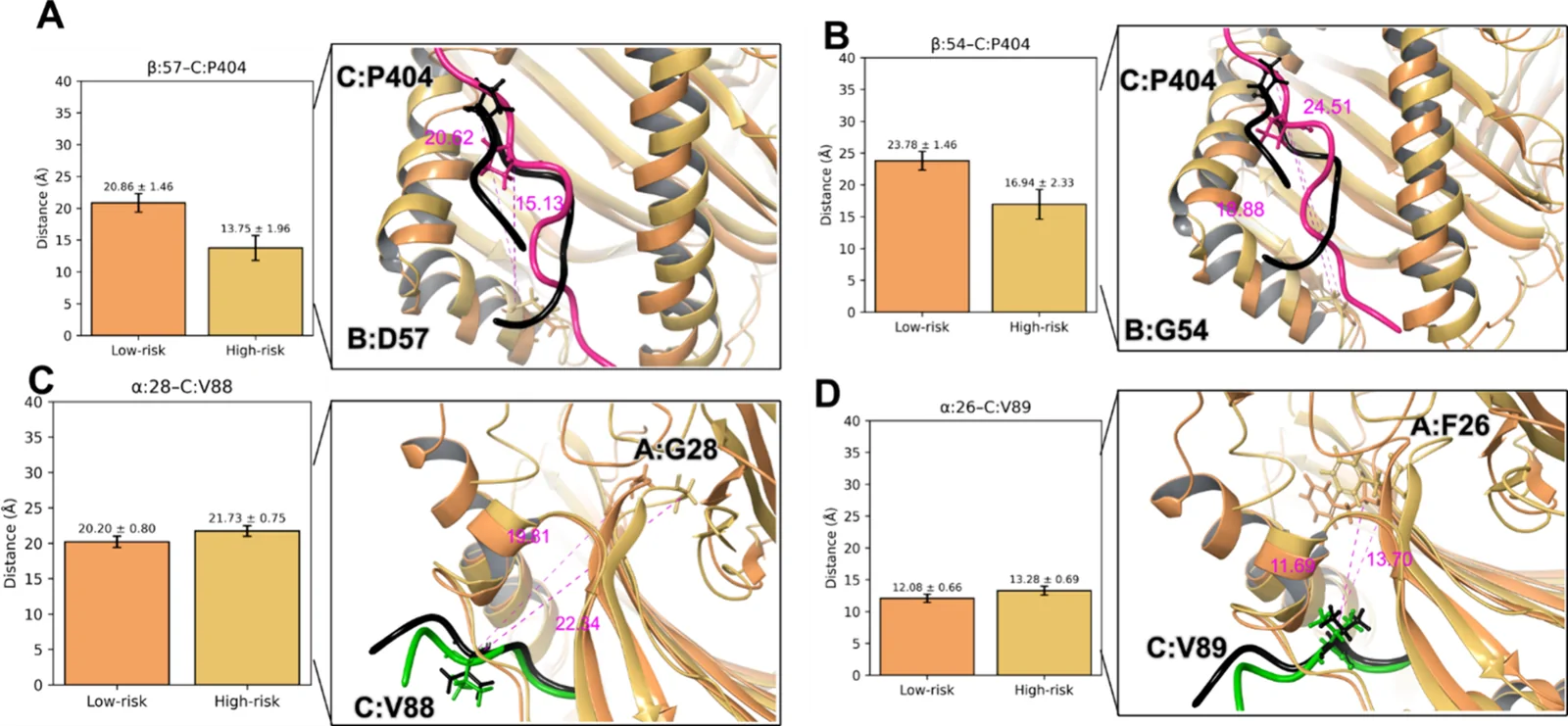
Discriminatory residue pairs at the pHLA interface and structural representation of the dominant frames. The graphs show the mean distances (± standard deviation) between residue pairs identified as the most informative by the ML models based on complexes with EBNA1_400–413_ and MBP_85–99_, comparing low- and high-risk alleles for MS. The corresponding structural representations illustrate these pairs at the pHLA interface for complexes containing EBNA1_400–413_ (β:57–C:P404 and β:54–C:P404 pairs) and MBP_85–99_ (α:28–C:V88 and α:26–C:V89 pairs). In the structural models, HLA-DRB1*15:01 is represented in yellow, whereas DRB1*15:02 is shown in orange. The EBNA1_400–413_ and MBP_85–99_ peptides are represented in pink and green, respectively. The distances between residues are indicated by magenta dashed lines, with values expressed in Å.

## 4 Discussion

The results of this study indicate that EBNA1_400–413_ does not reproduce the interaction mode of MBP_85–99_ at the pHLA interface, being incompatible with a mechanism of molecular mimicry as traditionally conceived. In this model, molecular mimicry assumes that an exogenous antigen shares sequence or structural similarity with an autoantigen, allowing cross-recognition by the TCR.^34^ In this sense, the mimetic peptide is expected to reproduce central aspects of pHLA complex organization, including the positioning of anchor residues in the HLA pockets and the exposure of residues compatible with TCR recognition, as demonstrated by structural studies of TCR–pMHC complexes.^88–93^

This model, however, does not adequately describe the behavior observed for EBNA1_400–413_ in the evaluated HLA-DRB1 alleles. Although this peptide remains associated with the binding groove throughout the simulations, its organization at the interface differs from that observed for MBP_85–99_. EBNA1_400–413_ shows a lower number of intermolecular contacts, greater solvent exposure, and a hydrogen-bond network that is more variable among the alleles, indicating accommodation that is less stabilized by recurrent contacts when compared with MBP_85–99_ and CLIP_103–117_. In addition, the MM/PBSA results show less favorable binding energy for EBNA1_400–413_, compared with MBP_85–99_ and CLIP_103–117_, suggesting a lower favorable energetic contribution to stabilization of the pHLA interface.

Despite these differences, EBNA1_400–413_ showed a particular behavior in the DRB1*15:01 and DRB1*15:03 alleles, both associated with higher MS risk. In these alleles, complexes containing EBNA1_400–413_ exhibited lower conformational variability and occupied low-energy regions that partially overlapped with those observed for complexes containing MBP_85–99_. This result indicates that the similarity between the complexes does not lie in the exact reproduction of peptide structure, the maintenance of the same inter-residue distances, or the preservation of the same interfacial contacts. Instead, it suggests that EBNA1_400–413_, when presented by DRB1*15:01 and DRB1*15:03, may modulate the pHLA interface in a way that favors access to and stabilization of conformational states close to those observed in complexes containing MBP_85–99_.

This interpretation is consistent with structural evidence demonstrating that TCR cross-reactivity does not require global structural equivalence between ligands. Different peptides can be recognized by the same TCR when they present distinct binding modes, provided that the pHLA complex exhibits conformational states compatible with formation of the recognition interface.^94^ More recently, it has been proposed that the conformational contribution may be determinant for cross-reactivity, since protein–protein interaction depends both on interfacial complementarity and on the distribution of accessible conformational states, even in the absence of detectable sequence or structural similarity.^95^ Thus, pHLA complexes with greater kinetic stability tend to persist longer as available ligands, increasing the opportunity for sampling by the TCR and, consequently, the probability of efficient recognition.^96^

The ML models reinforce this interpretation by demonstrating that MBP_85–99_ and EBNA1_400–413_ distinguish higher- and lower-risk alleles through distinct residue pairs at the pHLA interface. In the model based on MBP_85–‑99_, the most informative residue pairs were mainly concentrated in the α chain, especially at positions α:26-α:28, located within the P1 pocket. These positions are spatially adjacent to residue β:86, which exhibits a G/V dimorphism among the analyzed alleles, with valine in DRB1*15:01 and DRB1*15:03 and glycine in the remaining alleles. Thus, in complexes containing MBP_85–99_, allele discrimination is influenced by the local organization of the P1 pocket. In contrast, the model based on EBNA1_400–413_ identified a distinct set of discriminatory features, predominantly involving residues of the β chain, with emphasis on positions β:57 and β:54 in association with peptide residue C:P404. This shift in the regions that contribute most to classification indicates that EBNA1_400–413_ explores alternative regions of the β chain that also contribute to distinguishing higher-risk alleles from lower-risk alleles. Notably, two β-chain positions highlighted by our analyses were also independently associated with MS susceptibility in the HLA fine-mapping study by Patsopoulos et al. in 2013. β:57 showed a significant independent association, while β:86 was also independently associated with MS, with valine conferring increased risk relative to glycine.^97^ In addition, valine at position β:86 has been shown to affect both peptide binding and HLA-DRB1 αβ dimer stability,^98^ suggesting that variation at this position may influence antigen presentation and, consequently, the magnitude or specificity of the T-cell response to the bound peptide.

The combined MBP_85–99_ and EBNA1_400–413_ model revealed a point of convergence between the two systems. In this model, the most informative pairs were concentrated in the β:70−β:72 region, including the polymorphic site β:71. This position contains alanine in alleles of the DRB1*15 group and arginine in the DRB1*01:01, DRB1*11:01, and DRB1*16:01 alleles, although other HLA-DRB1 alleles may present different residues at this position, such as glutamine or lysine (Figure S18 in the Supporting Information). The association of β:71 with corresponding residues of the two peptides, especially MBP-C:F91 and EBNA1-C:F406, indicates that this region of the β chain contributes to distinguishing the allelic classes under both peptide conditions. However, this effect is not equivalent between ligands: differences involving β:71 are more pronounced in complexes with EBNA1_400–413_, whereas they remain more subtle in complexes with MBP_85–99_. Thus, β:71 does not define a spatial equivalence between the pairs formed with the two peptides, but rather identifies a region of the interface whose spatial organization is modulated in a peptide- and allele-dependent manner. The relevance of this position is further supported by HLA fine-mapping data, in which β:71 emerged as the most strongly associated DRβ1 amino acid position with MS susceptibility.^97^

The comparison among DRB1*15:01, DRB1*15:03, and DRB1*15:02 is particularly informative because it allows effects shared by the DRB1*15 group to be distinguished from those specifically associated with the higher-risk alleles. Alanine at β:71 is common to the three alleles, but only DRB1*15:01 and DRB1*15:03 contain β:V86, whereas DRB1*15:02 contains β:G86.^7^ This difference indicates that β:A71 alone is not sufficient to explain the lower conformational variability observed in EBNA1_400–413_ complexes with DRB1*15:01 and DRB1*15:03. The joint presence of β:A71 and β:V86 in these alleles may contribute to a more restricted organization of the pHLA interface, especially in the β:70−β:72 region and in the P1 pocket. In contrast, the presence of β:G86 in DRB1*15:02 may attenuate this effect, which is consistent with the greater conformational variability observed for EBNA1_400–413_ in this allele. On the other hand, the β:H30 substitution in DRB1*15:03 did not result in greater conformational variability similar to that observed in DRB1*15:02, suggesting that its effect on the interface is limited or compensated by the set of polymorphic residues, which, in the higher-risk alleles, contributes to the organization of the binding groove and the pHLA interface.

In addition to the global differences in dynamics and the discriminatory pairs identified by the models, the SASA and hydrogen-bond data suggest a local mechanism by which EBNA1_400–413_ may generate partial compatibility with MBP_85–99_. In EBNA1_400–413_, C:R402 showed high solvent exposure outside the terminal ends, whereas in MBP_85–99_, the highest SASA value was observed at C:K93. The analysis of the most favorable shared conformations in the FEL indicates that the N-terminal region of EBNA1_400–413_ undergoes rearrangement within the groove, repositioning the side chain of C:R402 to a region close to that occupied by C:K93 in MBP_85–99_. Because both residues carry a positive charge, this rearrangement may generate a local physicochemical similarity on the surface of the pHLA complex, even though the peptides do not share an equivalent global pose.

This rearrangement may be favored by the local organization of the β:70−β:72 region. In DRB1*15 alleles, alanine at β:71 contributes to a more spacious P4 pocket, whereas bulkier residues at this position, such as arginine, can restrict the available space and modify the local geometry and physicochemical properties of the peptide-binding groove.^20,99^ This difference is compatible with a distinct local accommodation of EBNA1_400–413_, particularly involving C:R403 in the P4 region, and with the repositioning of C:R402 toward the region occupied by C:K93 in MBP_85–99_. In addition, the hydrogen-bond pattern observed in the risk alleles reinforces this interpretation. Although EBNA1_400–413_ shows more variable hydrogen-bond networks among alleles, recurrent interactions involving C:G410−β:D57 at P9, C:P408−β:W61 at P7, and C:R403−β:D28 at P4 were observed in complexes with DRB1*15:01 and DRB1*15:03. These interactions indicate that, in these alleles, EBNA1_400–413_ maintains recurrent contacts in specific regions of the groove. Thus, the lower conformational variability of EBNA1_400–413_ in DRB1*15:01 and DRB1*15:03 may result from the combination of the local geometry of the β:70−β:72 region, the presence of β:V86, recurrent interactions at P4, P7, and P9, and the solvent exposure of positively charged residues potentially relevant to the recognition interface (Figure 11).

**Figure 11:**
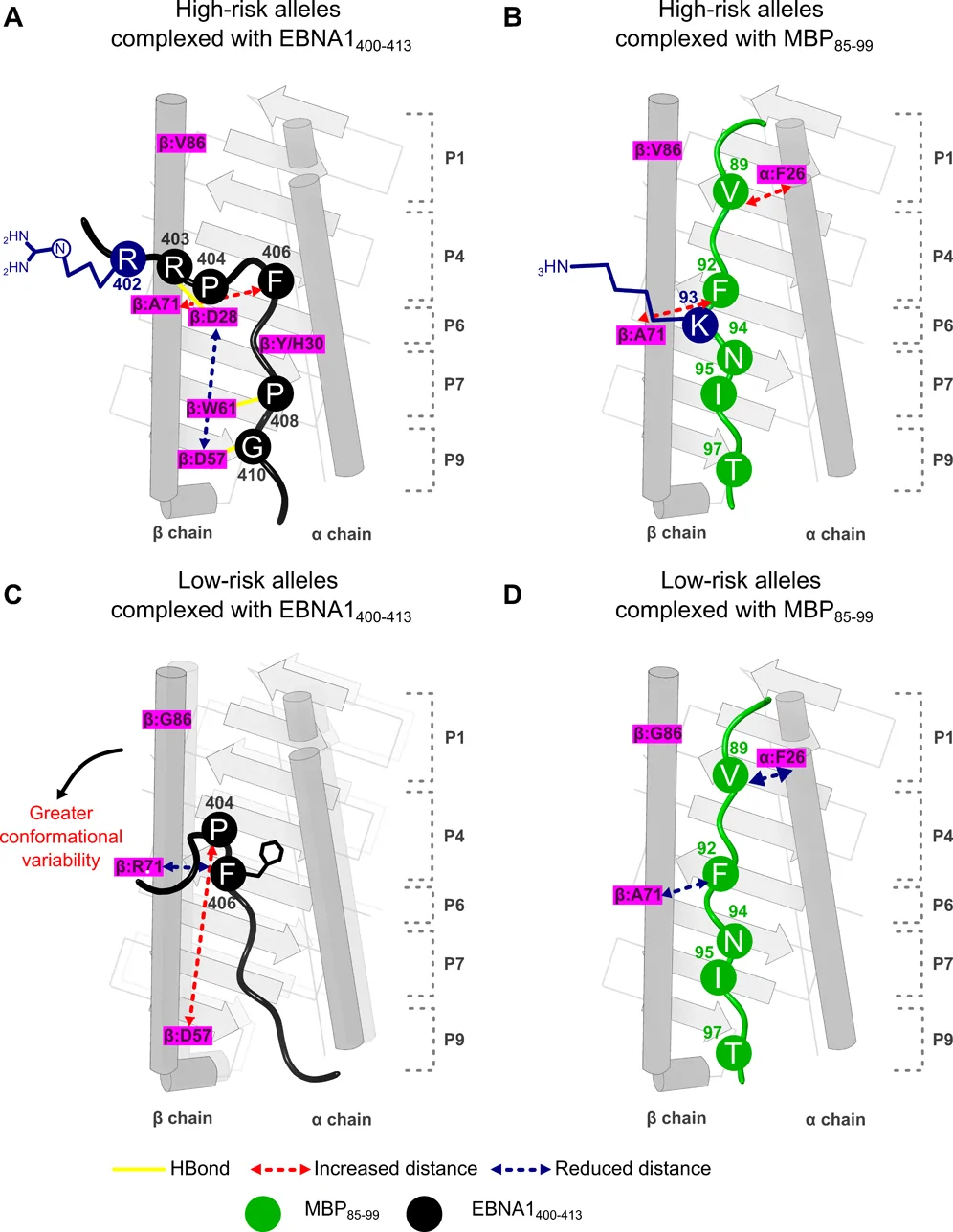
Proposed model for functional mimicry dependent on the dynamics of the pHLA interface between EBNA1_400–413_ and MBP_85–99_. The figure schematizes local differences in the organization of the EBNA1_400–413_ and MBP_85–99_ peptides within the HLA-DRB1 binding groove and proposes a mechanism of partial compatibility between the complexes. (A) In higher-risk alleles, EBNA1_400–413_ is accommodated in the groove with recurrent contacts in specific regions, including interactions involving C:R403 in the P4 region, C:P408 at P7, and C:G410 at P9. The joint presence of β:A71 and β:V86 may favor a more restricted local organization of the interface, allowing the repositioning of C:R402 to an exposed region on the surface of the pHLA complex. (B) In the same risk alleles, MBP_85–99_ maintains a more conserved binding mode, with anchor residues accommodated in the main pockets of the groove and exposure of C:K93 on the surface of the complex. The spatial proximity between C:R402 of EBNA1_400–413_ and C:K93 of MBP_85–99_ suggests a local physicochemical similarity, since both residues are positively charged. (C) In lower-risk alleles, the presence of different residues at critical positions of the β chain, such as β:R71 and β:G86, may modify the local geometry of the interface and result in greater conformational variability. (D) In contrast, MBP_85–99_ preserves a more conserved binding mode within the groove, even in lower-risk alleles, although without reproducing the same distances between C:V89−α:F26 and C:F92−β:A71 observed in the risk alleles.

The less favorable binding energy estimated for EBNA1_400–413_ compared with MBP_85–99_ and CLIP_103–117_ indicates a lower energetic contribution to stabilization of the pHLA interface within the comparative framework used here. Experimental studies have independently demonstrated efficient binding and loading of MBP-derived peptides onto HLA-DRB1 molecules, including DRB1*15:01, and have extensively characterized CLIP binding and HLA-DM-mediated peptide exchange.^14,27,100^ In contrast, although EBNA1_400–413_ has been investigated in previous structural and computational studies, direct experimental measurements of its binding affinity and loading kinetics across the HLA-DRB1 alleles evaluated here remain limited. In this context, the present results provide a comparative energetic characterization of EBNA1_400–413_ across alleles with distinct MS susceptibility profiles and indicate that its interaction with the HLA-DRB1 groove differs energetically from those of MBP_85–99_ and CLIP_103–117_. Importantly, however, peptide selection by MHC-II is not determined solely by the energetics of the final pHLA complex. HLA-DM catalyzes CLIP release and peptide exchange and edits the presented repertoire by favoring pMHC-II complexes with sufficient kinetic stability to persist after loading. Experimental studies further demonstrate that peptide–MHC-II stability and HLA-DM-mediated exchange are not necessarily coupled in a simple manner.^14,27^ Accordingly, it is plausible that EBNA1_400–413_, despite its less favorable binding energy, may still be presented by DRB1*15:01 and DRB1*15:03, particularly because complexes formed with these alleles exhibited lower conformational variability in our simulations, indicating a more restricted conformational ensemble once the pHLA complex is formed.

The results obtained in this study support a model of functional mimicry dependent on the dynamics of the pHLA interface. In this model, EBNA1_400–413_ does not mimic MBP_85–99_ through global structural similarity, nor through the exact reproduction of the same distances or interfacial contacts. Instead, in the DRB1*15:01 and DRB1*15:03 alleles, the combination of β:A71 and β:V86, recurrent hydrogen-bond interactions at P4, P7, and P9, and the repositioning of C:R402 of EBNA1_400–413_ to a region close to that occupied by C:K93 in MBP_85–99_ may favor conformational states with lower variability and partial compatibility between the two complexes. Thus, β:A71 and β:V86 may act as important components of the dynamic stabilization of complexes with EBNA1_400–413_ in risk-associated alleles, but their contribution should be interpreted together with the other polymorphisms that define each allele. Because the TCR was not explicitly included in the simulated systems, the present study does not directly resolve receptor engagement or cross-reactivity. Nevertheless, the conformational states and physicochemical organization of the exposed pHLA surface define the molecular context presented to the TCR, and the partial compatibility identified here provides insights into how distinct pHLA complexes may access conformational states compatible with recognition. Accordingly, the main contribution of this study is to show that functional mimicry may emerge from allele-dependent dynamics and local organization of the pHLA interface rather than from ideal structural equivalence between the ligands.

## Supplementary Material



## Data Availability

The datasets generated and analyzed during the current study are not publicly available but are available from the corresponding author upon reasonable request.

## Notes

Figure S1, RMSD profiles of the HLA ectodomain and bound peptide across independent molecular dynamics simulation replicas. Figure S2, area per lipid values for the membrane components across the molecular dynamics simulations. Figure S3, RMSD values of the pHLA interface across molecular dynamics simulation replicas. Figure S4, radius of gyration (*R*_g_) values of the pHLA interface across molecular dynamics simulation replicas. Figure S5, Pairwise RMSD matrices between frames from molecular dynamics trajectories of pHLA complexes containing the peptides CLIP_103–117_, MBP_85–99_, and EBNA1_400–413_. Figure S6, secondary structure trajectory for HLA-DRB1 alleles in complex with CLIP_103–117_ over 500 ns of MD simulation. Figure S7, secondary structure trajectory for HLA-DRB1 alleles in complex with MBP_85–99_ over 500 ns of MD simulation. Figure S8, secondary structure trajectory for HLA-DRB1 alleles in complex with EBNA1_400–413_ over 500 ns of MD simulation. Figure S9, temporal evolution of the number of contacts at the pHLA interface during the molecular dynamics trajectories for HLA-DRB1 complexes bound to the peptides MBP_85–99_, EBNA1_400–413_, and CLIP_103–117_. Figure S10, SASA values along the sequence of the α1 and β1 domains and of the peptides bound to different HLA-DRB1 alleles. Figure S11, hydrogen-bonding networks at the pHLA interface for the MBP_85–99_ peptide across different HLA-DRB1 alleles. Figure S12, hydrogen-bonding networks at the pHLA interface for the EBNA1_400–413_ peptide across different HLA-DRB1 alleles. Figure S13, per-residue binding free-energy decomposition obtained by MM/PBSA for HLA-DRB1 complexes associated with the peptides CLIP_103–117_, MBP_85–99_, and EBNA1_400–413_. Figure S14, free-energy landscapes (FELs) of the pHLA complexes projected onto PC1 and PC2 for HLA-DRB1 alleles under the MBP_85–99_ and EBNA1_400–413_ conditions. Figure S15, performance of Random Forest models trained using residue pairs derived from interactions in pHLA complexes. Figure S16, mean distances of discriminative residue pairs involving position β:71 at the pHLA interface. Figure S17, structural comparison of the dominant frames of pHLA complexes highlighting residue pairs at the interface. Figure S18, multiple alignment of different HLA-DRB1 alleles. Table S1, number of low-energy regions (≤ 2.5 kJ/mol) identified in the free-energy landscapes (FEL) projected onto the PC1–PC2 space for pHLA complexes containing MBP_85–99_ and EBNA1_400–413_ associated with different HLA-DRB1 alleles. Table S2, performance of Random Forest models, evaluated using ten-fold cross-validation, trained on inter-residue distances at the pHLA interface to classify high-risk alleles (DRB1*15:01 and DRB1*15:03) and low-risk alleles (DRB1*01:01, DRB1*11:01, DRB1*15:02, DRB1*16:01). Table S3, classification performance of the final Random Forest models using pHLA inter-residue distances for discrimination between high-risk and low-risk HLA-DRB1 alleles across different peptide conditions. Table S4, pHLA interface residue pairs with the highest contribution to the classification of HLA-DRB1 alleles associated with lower risk of MS, as identified by machine learning (ML) models (PDF)
