# Supplementary material for: HLA-DRB1 Polymorphisms Modulate the Dynamic Compatibility between the Presentation of EBNA1400–413 and MBP85–99
Source: ACS Omega. 2026 Sep 17;11(38):57521–40. doi: 10.1021/acsomega.6c06047 (PMC13629357; doi:10.1021/acsomega.6c06047)
Supplement: Supplementary file 1 [file ao6c06047_si_001.pdf]

Supplementary Material

HLA-DRB1 polymorphisms modulate the dynamic compatibility between the presentation of $EBNA1_{400-413}$ and MBP_{85-99}

Levy Bueno Alves[1], Silvana Giuliatti[1*]

[1] Department of Genetics, Ribeirão Preto Medical School, University of São Paulo, USP

*** Correspondence:** silvana@fmrp.usp.br

///

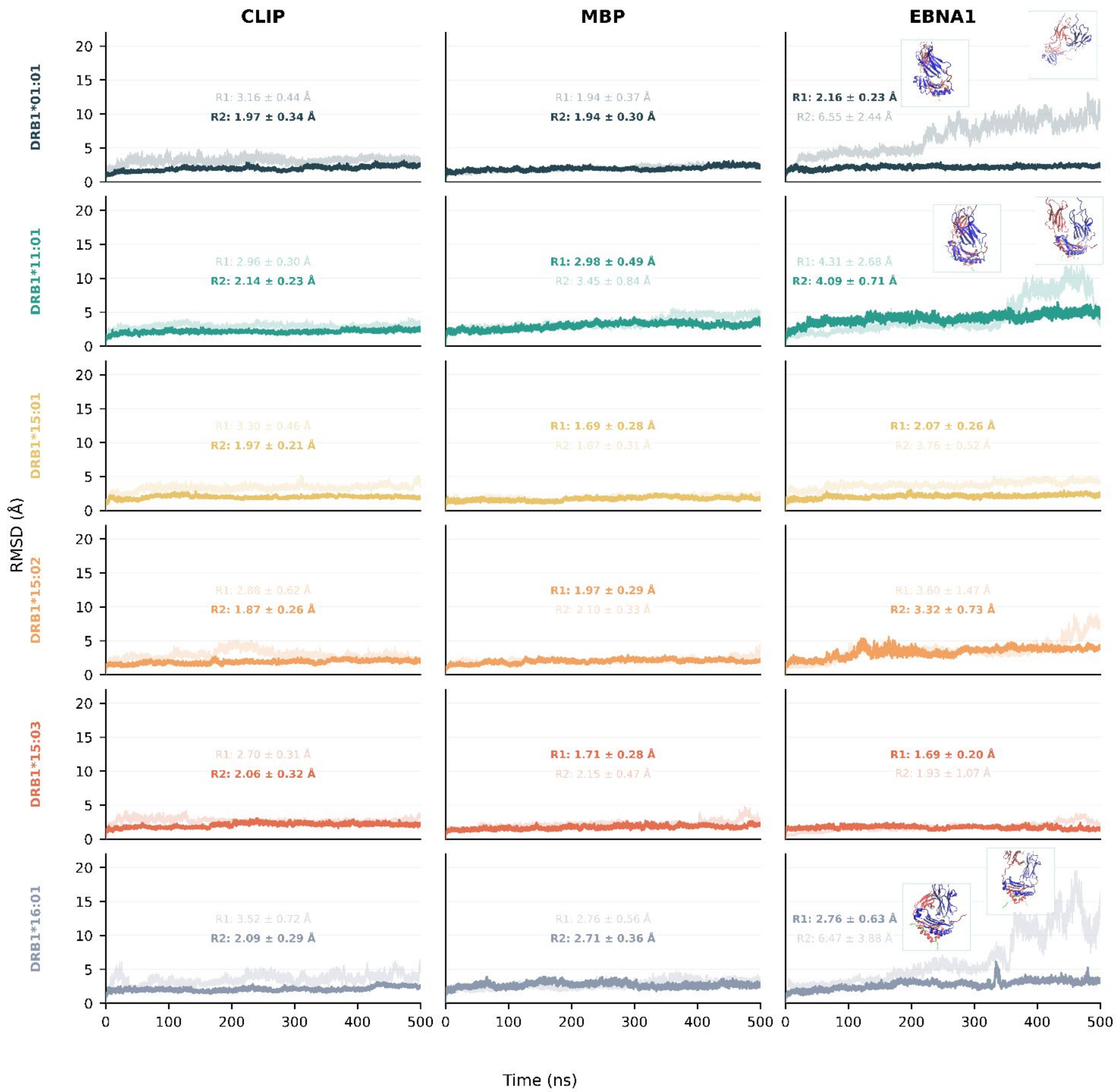

Figure S1. RMSD profiles of the HLA ectodomain and bound peptide across independent molecular dynamics simulation replicas. RMSD was calculated over the 500 ns production trajectories considering the backbone atoms of the HLA ectodomain together with the bound peptide for complexes containing $CLIP_{103\text{-}117}$, $MBP_{85\text{-}99}$, and $EBNA1_{400\text{-}413}$, shown in columns, and the six HLA-DRB1 alleles, shown in rows. Both replicas are displayed for each system. The replica selected for subsequent analyses is represented by the opaque line, whereas the alternative replica is shown with increased transparency. Mean RMSD values and corresponding standard deviations (mean ± SD) are indicated within each panel for both replicas, following the same transparency scheme. Representative structural frames are shown for selected trajectories exhibiting pronounced increases in RMSD, illustrating conformational rearrangements predominantly involving the α2 and β2 domains of the HLA ectodomain. These structures illustrate the origin of the larger global RMSD values observed in these replicas and distinguish such ectodomain rearrangements from changes localized at the peptide-binding interface.

/

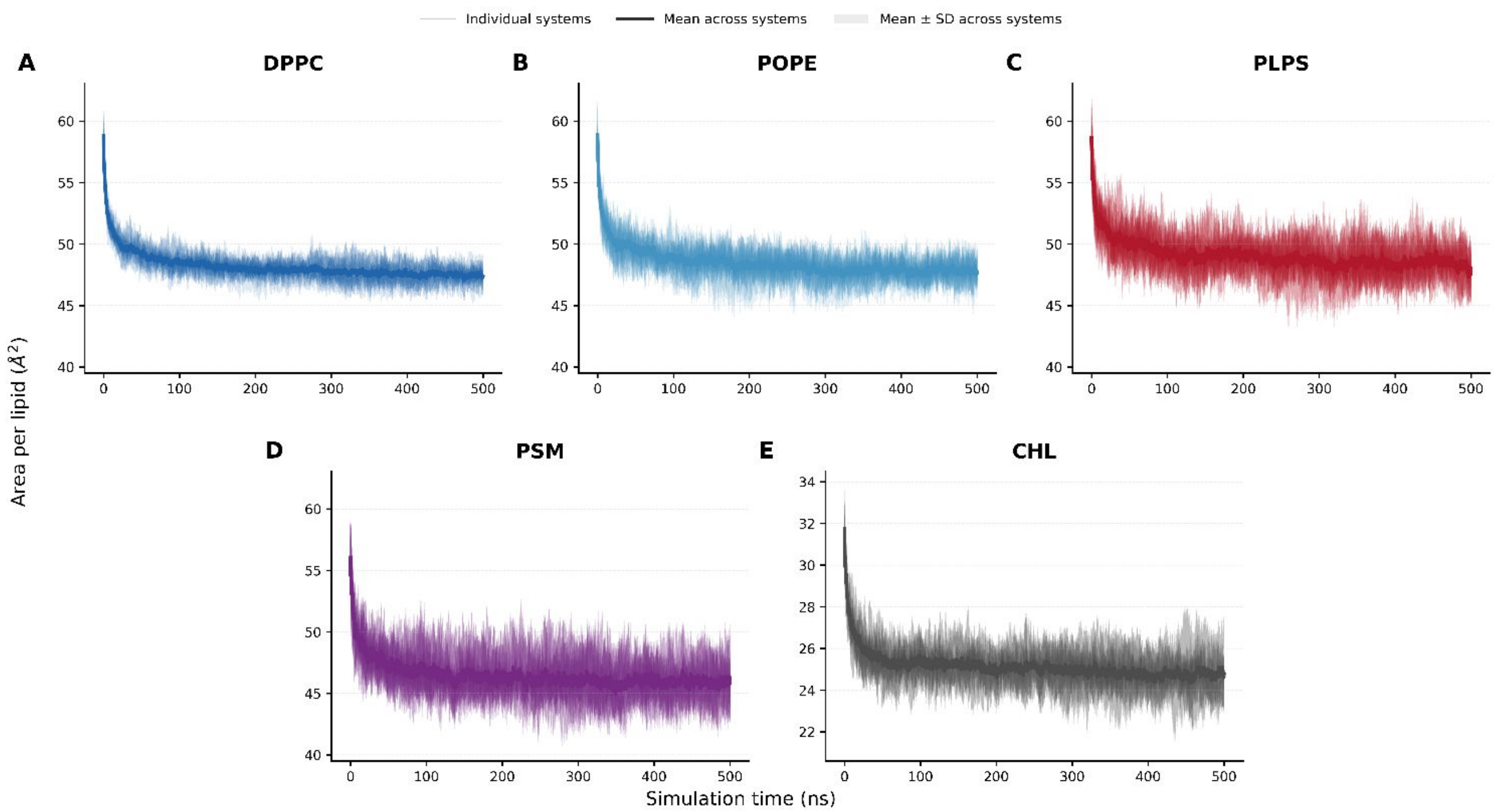

Figure S2. Area per lipid values for the membrane components across the molecular dynamics simulations. Area per lipid was monitored over the 500 ns production trajectories for (A) DPPC, (B) POPE, (C) PLPS, (D) PSM, and (E) cholesterol (CHL) across the 18 simulated systems. Thin lines represent the individual systems, the solid line represents the mean across systems, and the shaded region indicates the mean ± standard deviation. All lipid species showed an initial decrease in area per lipid followed by stabilization, with comparable profiles among the simulated systems throughout most of the trajectories.

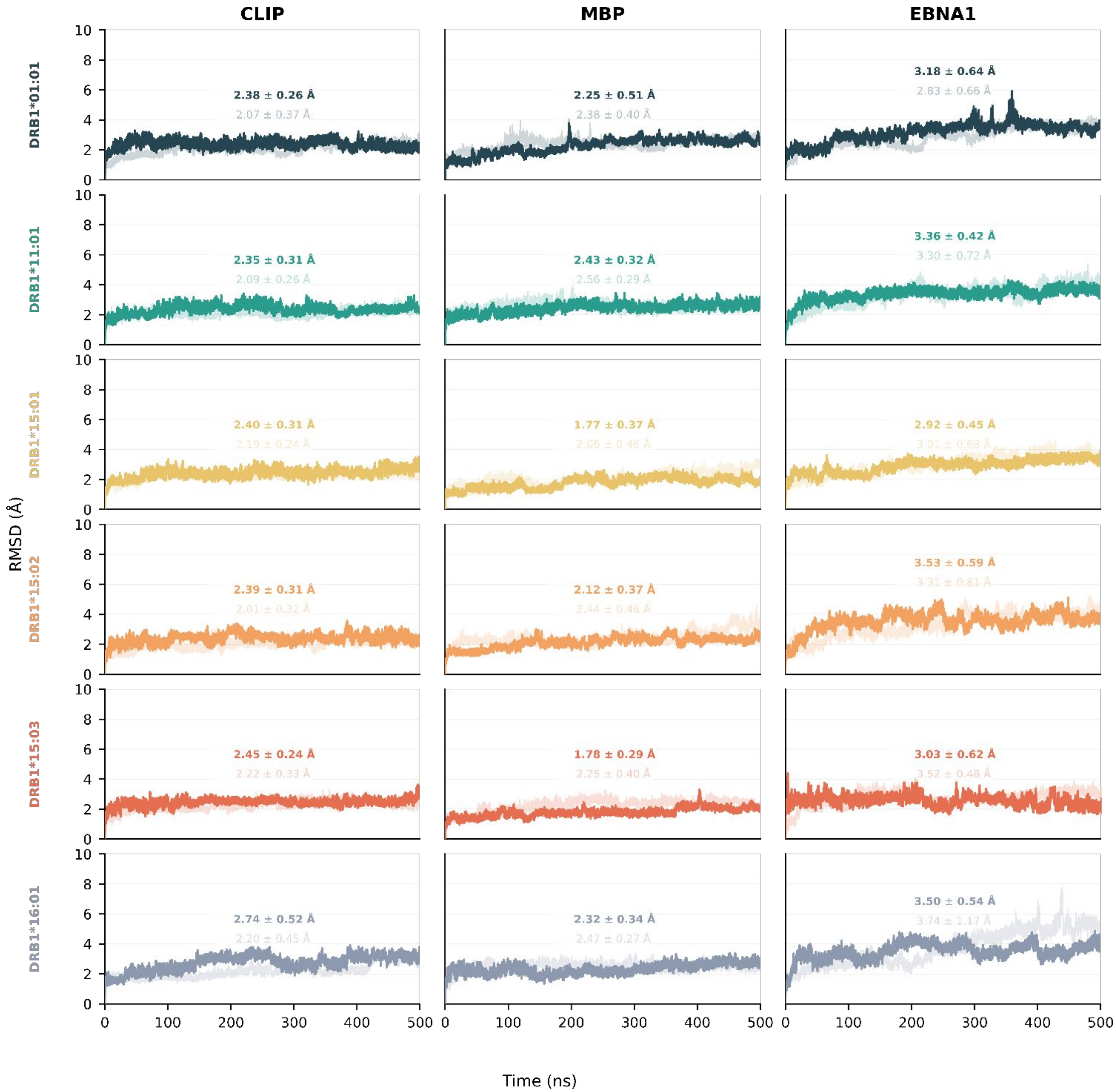

Figure S3. RMSD values of the pHLA interface across molecular dynamics simulation replicas. RMSD was calculated over the 500 ns production simulations using the backbone atoms of the HLA peptide-binding groove together with the bound peptide for complexes containing $CLIP_{103\text{-}117}$, $MBP_{85\text{-}99}$, and $EBNA1_{400\text{-}413}$, shown in columns, and the six HLA-DRB1 alleles, shown in rows. Both replicas are displayed for each system. The replica previously selected for subsequent analyses based on the RMSD behavior of the HLA ectodomain together with the bound peptide (Supplementary Figure 1) is represented by the opaque line, whereas the alternative replica is shown with increased transparency. This comparison illustrates the local conformational behavior of the peptide-binding interface across the simulations.

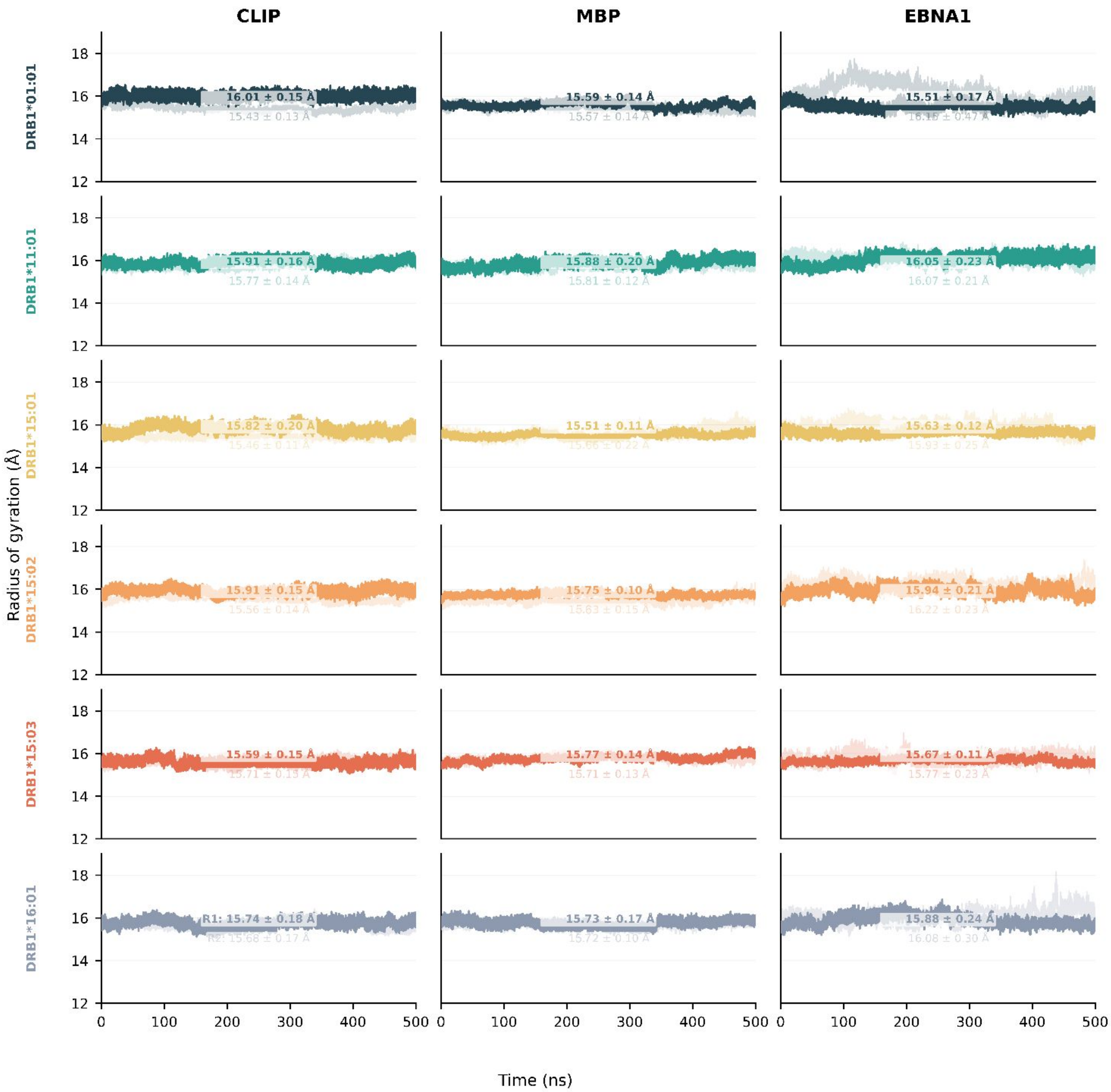

Figure S4. Radius of gyration (Rg) values of the pHLA interface across molecular dynamics simulation replicas. Rg was calculated over the 500 ns production simulations for complexes containing $CLIP_{103-117}$, MBP_{85-99}, and $EBNA1_{400-413}$, shown in columns, and the six HLA-DRB1 alleles, shown in rows. Both independent replicas are displayed for each system. The replica previously selected for subsequent analyses based on the ectodomain RMSD assessment shown in Supplementary Figure S1 is represented by the opaque line, whereas the alternative replica is shown with increased transparency. Mean Rg values and corresponding standard deviations (mean ± SD) are indicated within each panel.

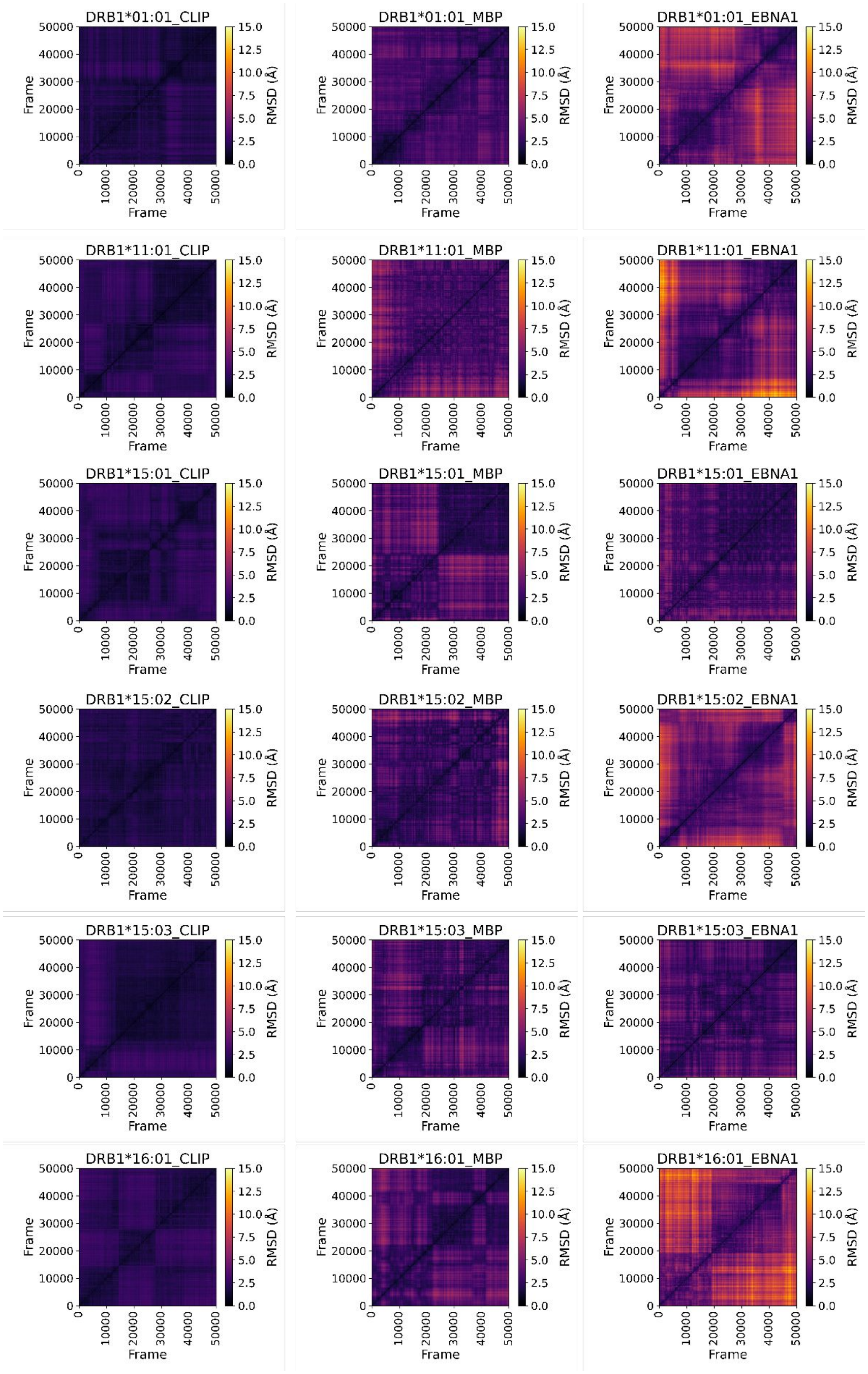

Figure S5. Pairwise RMSD matrices between frames from molecular dynamics trajectories of pHLA complexes containing the peptides $CLIP_{103\text{-}117}$, $MBP_{85\text{-}99}$, and $EBNA1_{400\text{-}413}$.

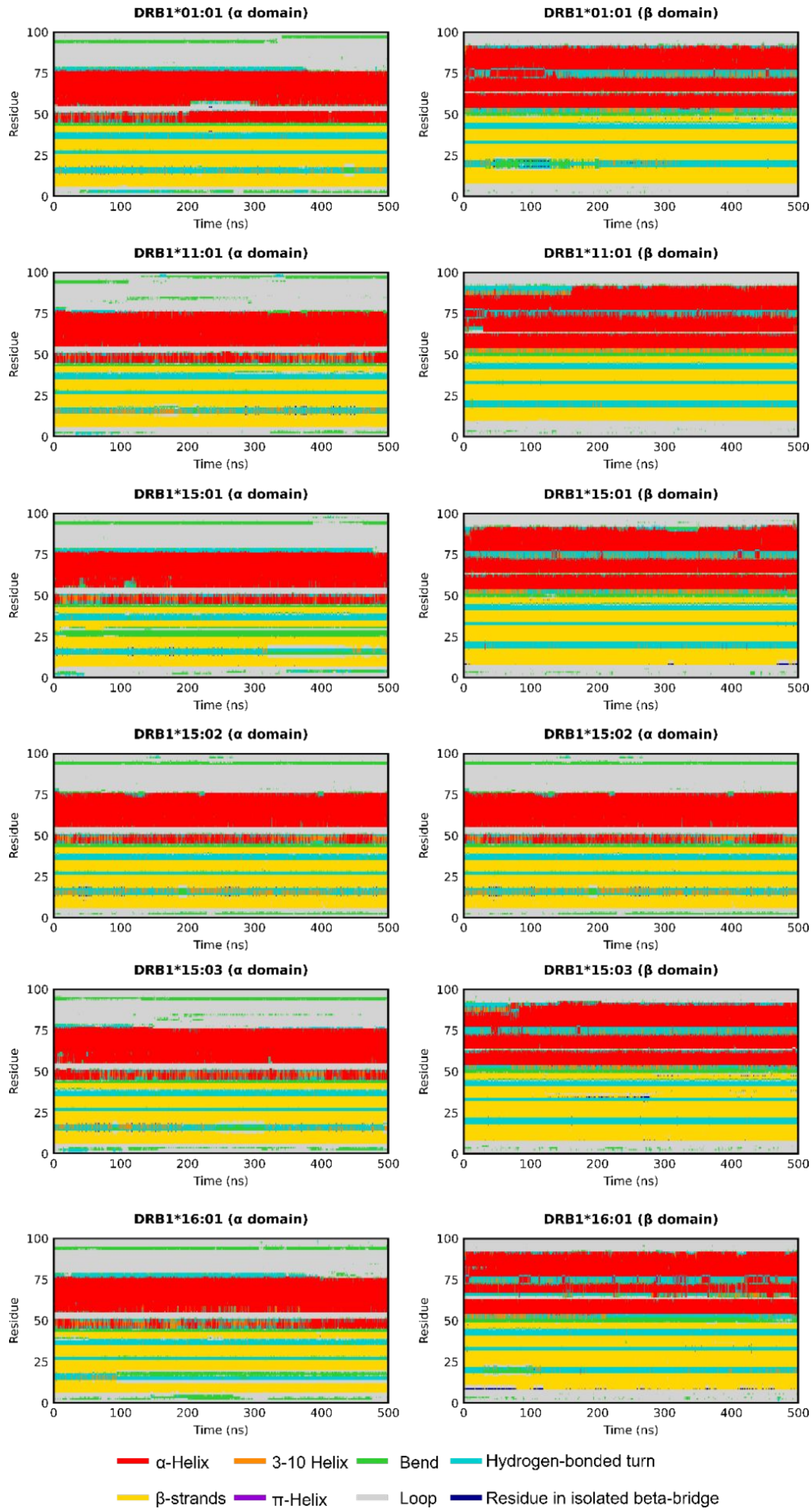

Figure S6. Secondary structure trajectory for HLA-DRB1 alleles in complex with $CLIP_{103–117}$ over 500 ns of MD simulation.

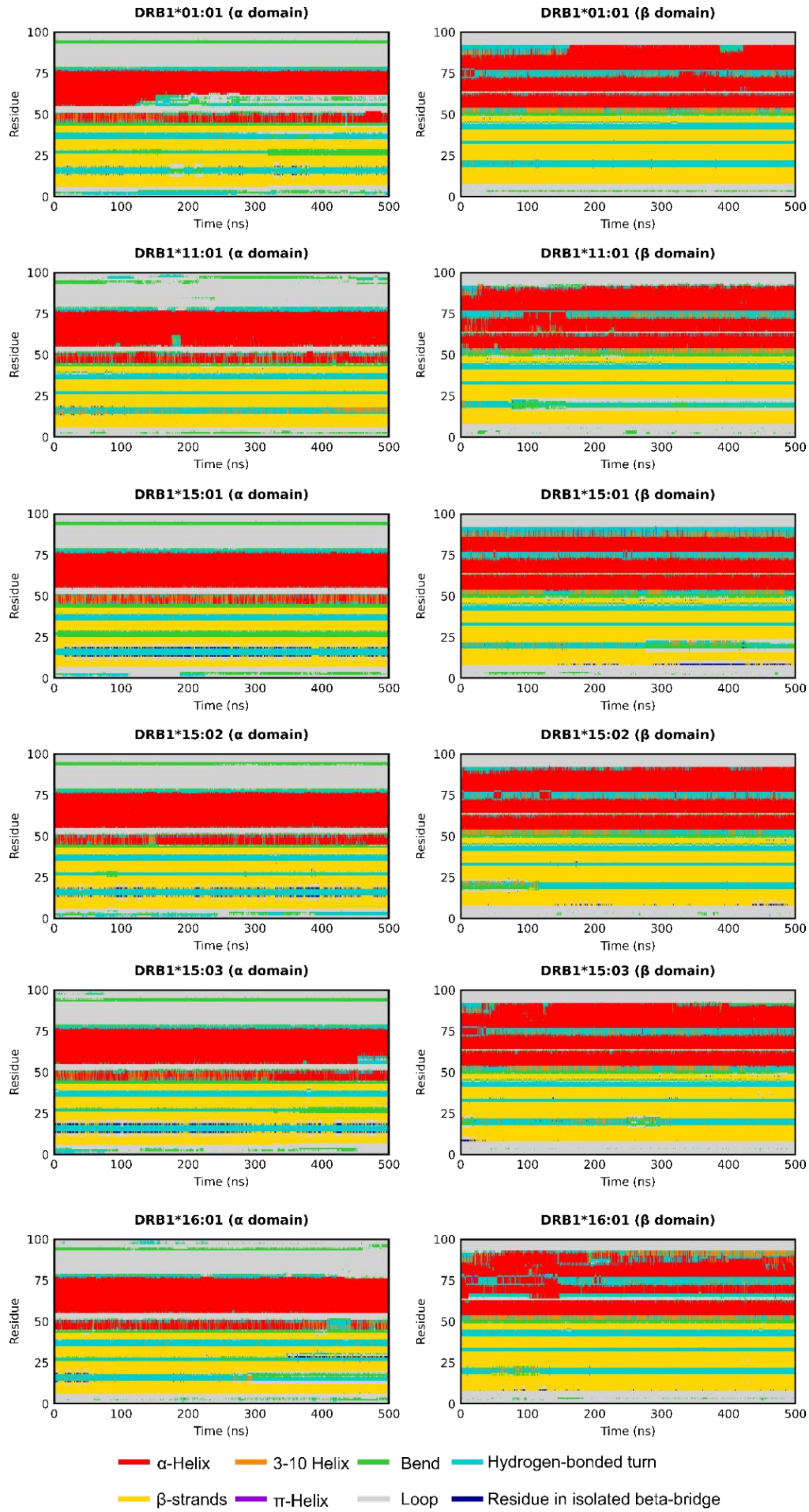

Figure S7. Secondary structure trajectory for HLA-DRB1 alleles in complex with $MBP_{85–99}$ over 500 ns of MD simulation.

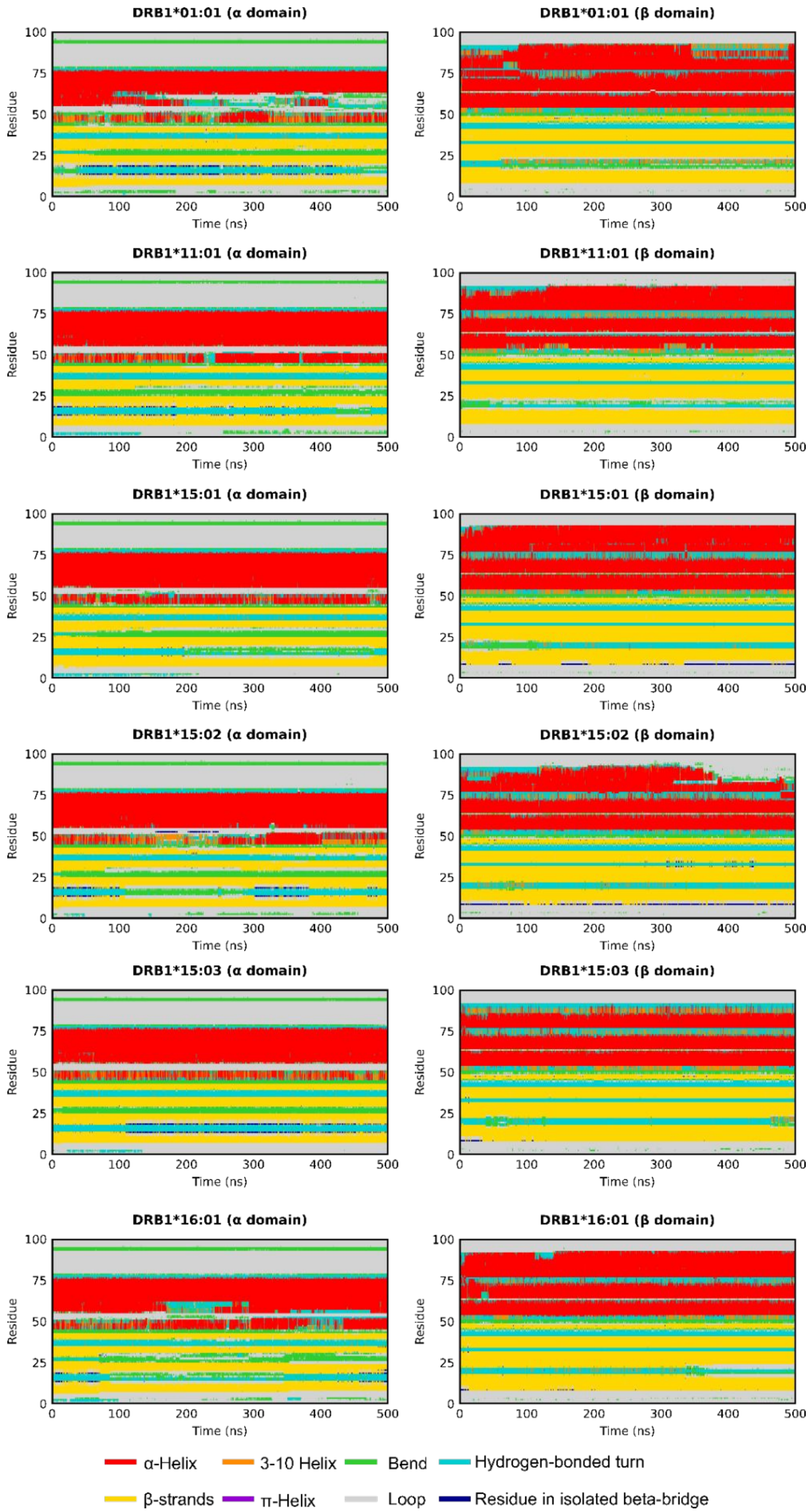

Figure S8. Secondary structure trajectory for HLA-DRB1 alleles in complex with EBNA1$_{400–413}$ over 500 ns of MD simulation.

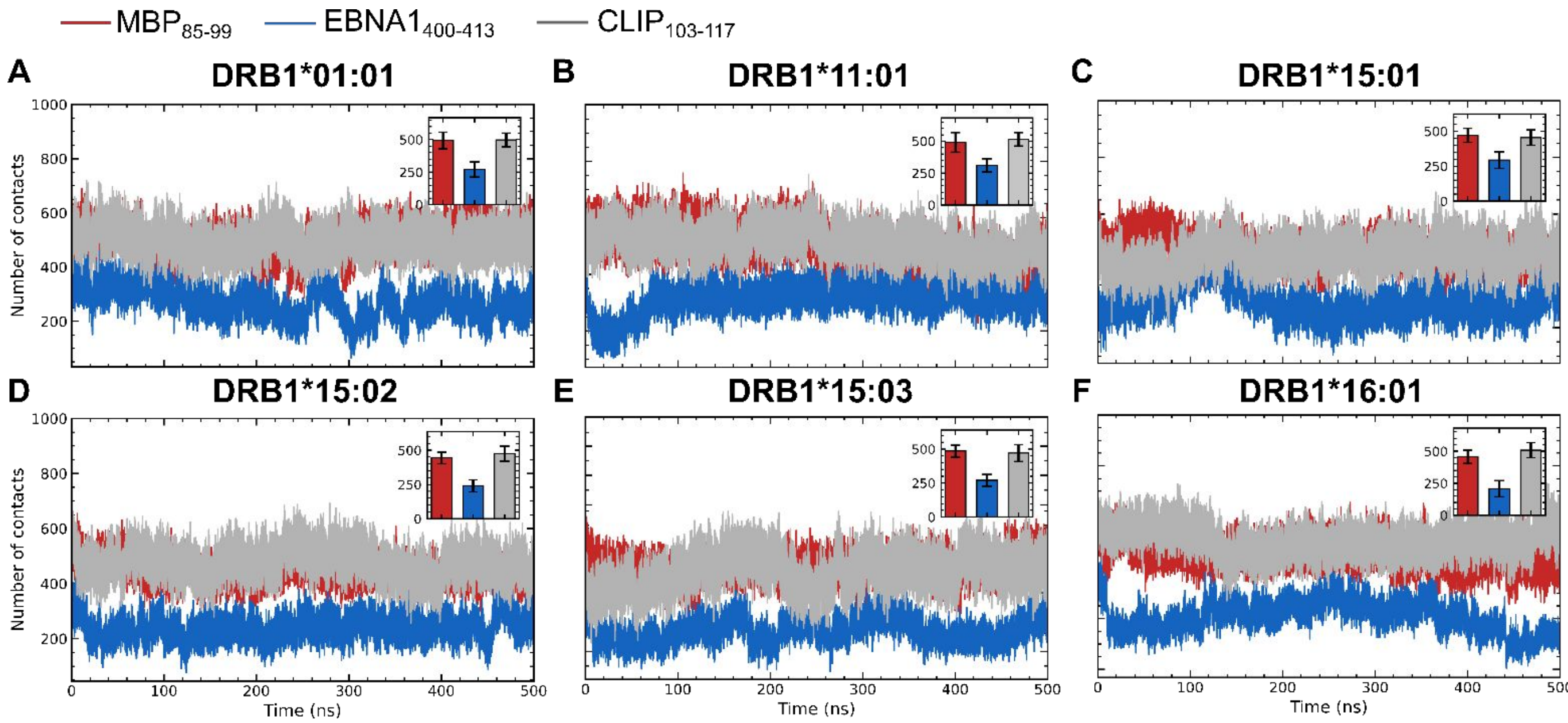

Figure S9. Temporal evolution of the number of contacts at the pHLA interface during the molecular dynamics trajectories for HLA-DRB1 complexes bound to the peptides MBP_{85-99}, $EBNA1_{400-413}$, and $CLIP_{103-117}$. Contacts were calculated using a cutoff distance of 3.5 Å between heavy atoms of peptide residues and residues in the HLA binding groove. Each panel corresponds to a distinct HLA-DRB1 allele: (A) DRB1*01:01, (B) DRB1*11:01, (C) DRB1*15:01, (D) DRB1*15:02, (E) DRB1*15:03, and (F) DRB1*16:01. The inset graphs show the mean contact values for each condition, accompanied by the corresponding standard deviation.

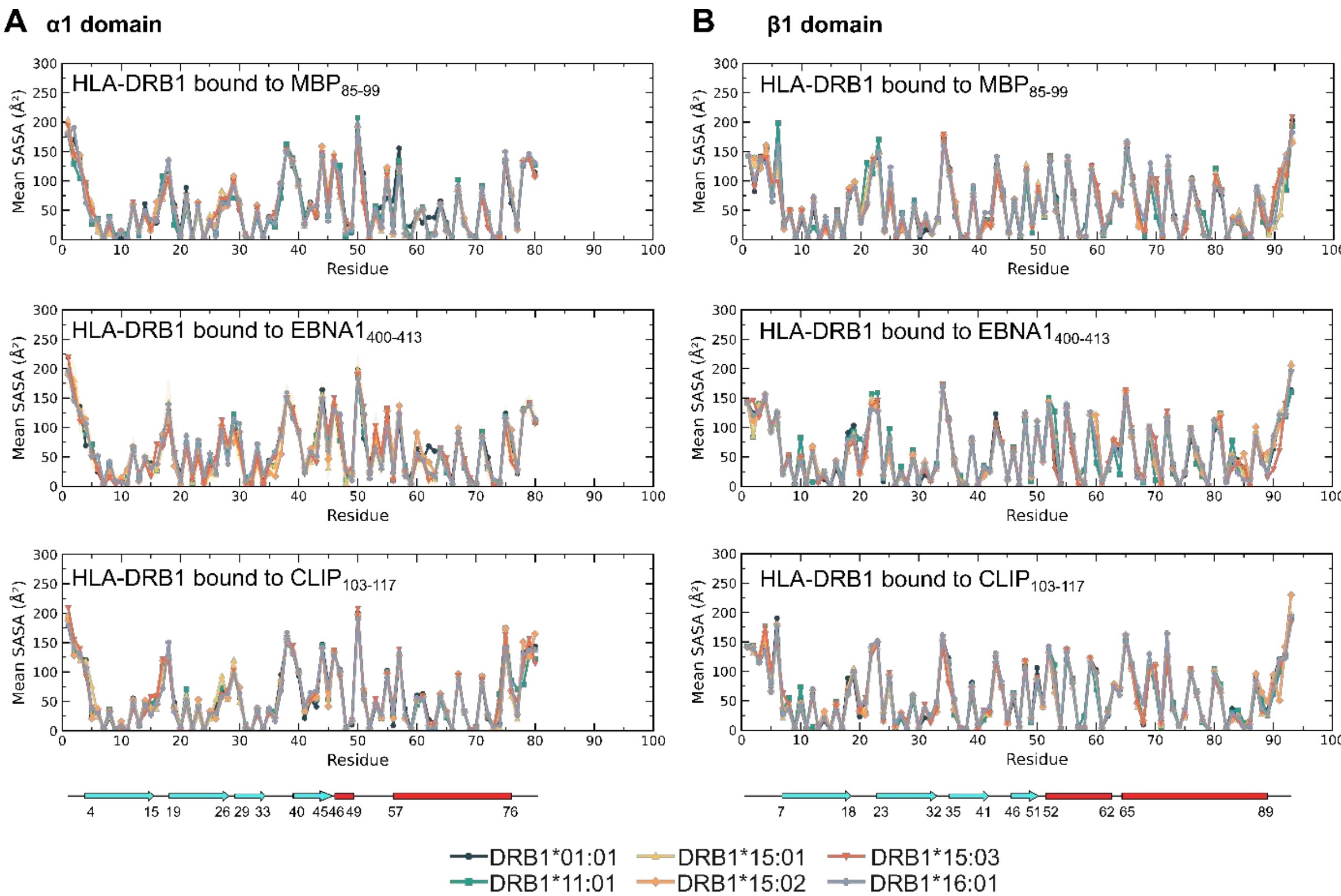

Figure S10. SASA values along the sequence of the α1 and β1 domains and of the peptides bound to different HLA-DRB1 alleles. The panels (A–B) show the SASA profiles of the binding groove (α1/β1) for $MBP_{85–99}$, $EBNA1_{400-413}$, and $CLIP_{103–117}$. The secondary structure of the α1 and β1 domains is indicated along the sequence, with β-strands (cyan) and α-helices (red).

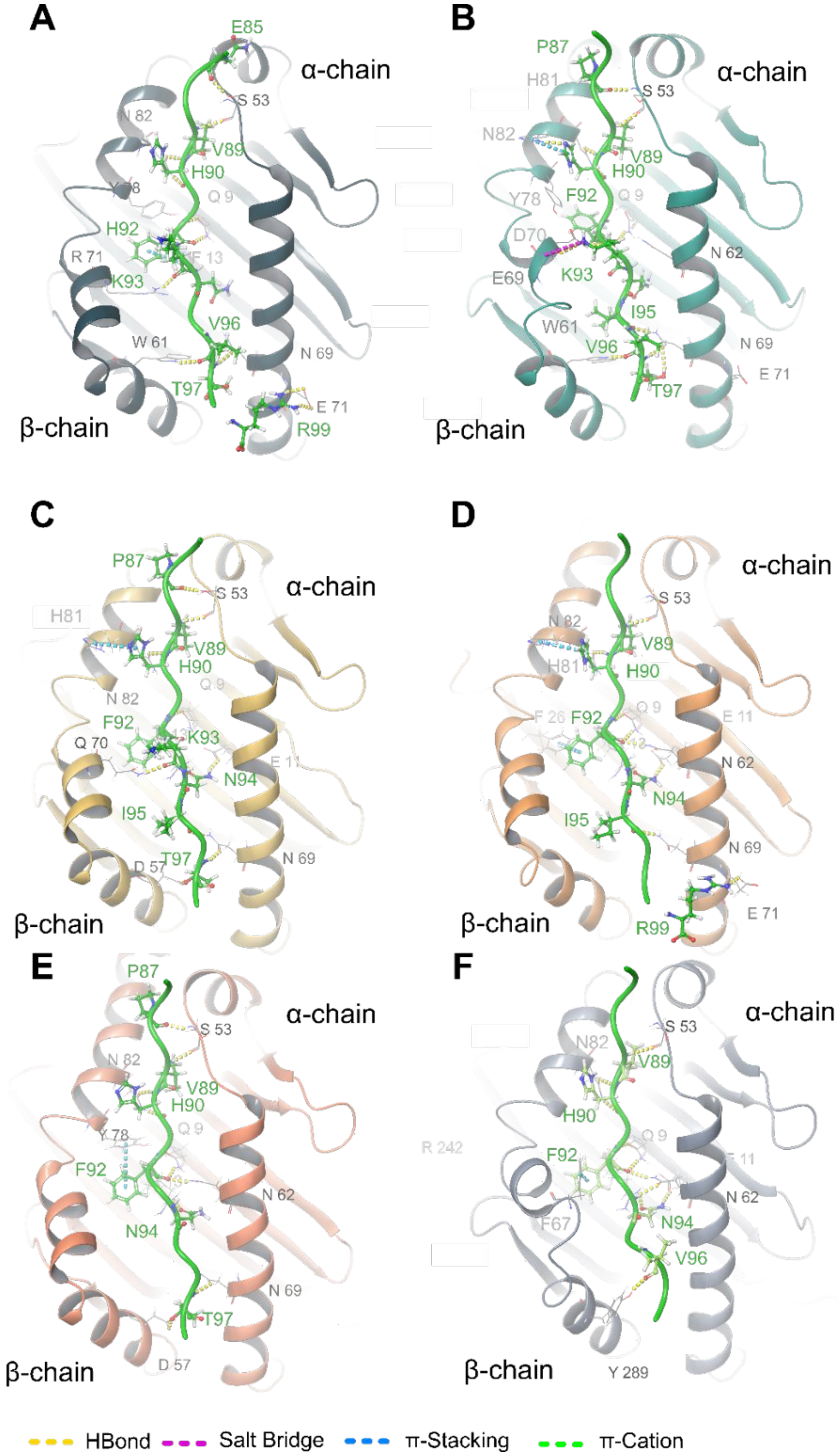

Figure S11. Hydrogen-bonding networks at the pHLA interface for the $MBP_{85–99}$ peptide across different HLA-DRB1 alleles. Structural representations of the dominant pose of the pHLA complexes, obtained from molecular dynamics trajectory analysis, showing the peptide (green) accommodated within the binding groove formed by the α and β chains of HLA-DR (cartoon representation). Hydrogen bonds are indicated by dashed lines, and the residues involved in the interactions are labeled. (A) DRB1*01:01; (B) DRB1*11:01; (C) DRB1*15:01; (D) DRB1*15:02; (E) DRB1*15:03; (F) DRB1*16:01.

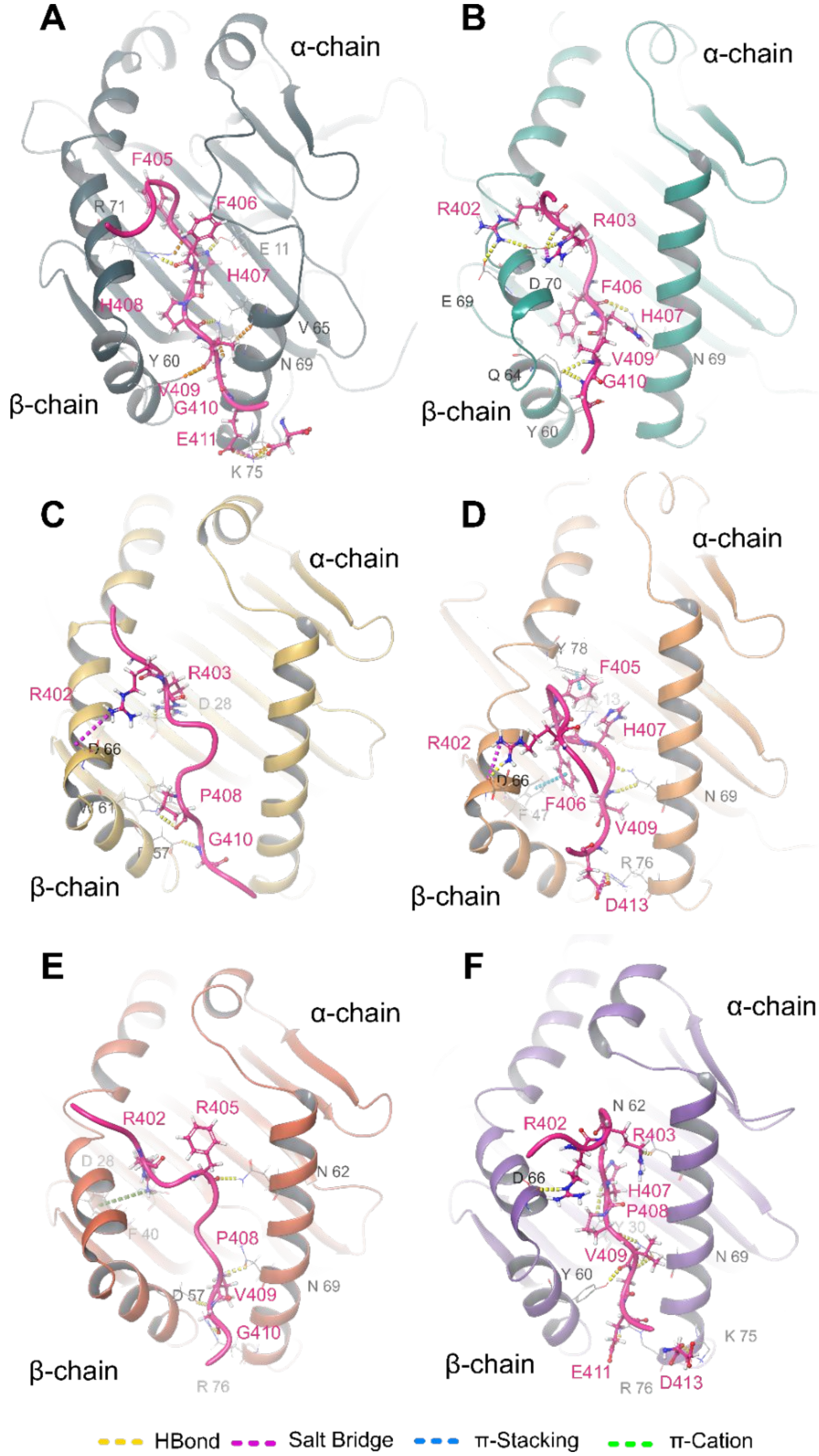

Figure S12. Hydrogen-bonding networks at the pHLA interface for the EBNA1$_{400-413}$ peptide across different HLA-DRB1 alleles. Structural representations of the dominant pose of the pHLA complexes, obtained from molecular dynamics trajectory analysis, showing the peptide (pink) accommodated within the binding groove formed by the α and β chains of HLA-DR (cartoon representation). Hydrogen bonds are indicated by dashed lines, and the residues involved in the interactions are labeled. (A) DRB1*01:01; (B) DRB1*11:01; (C) DRB1*15:01; (D) DRB1*15:02; (E) DRB1*15:03; (F) DRB1*16:01.

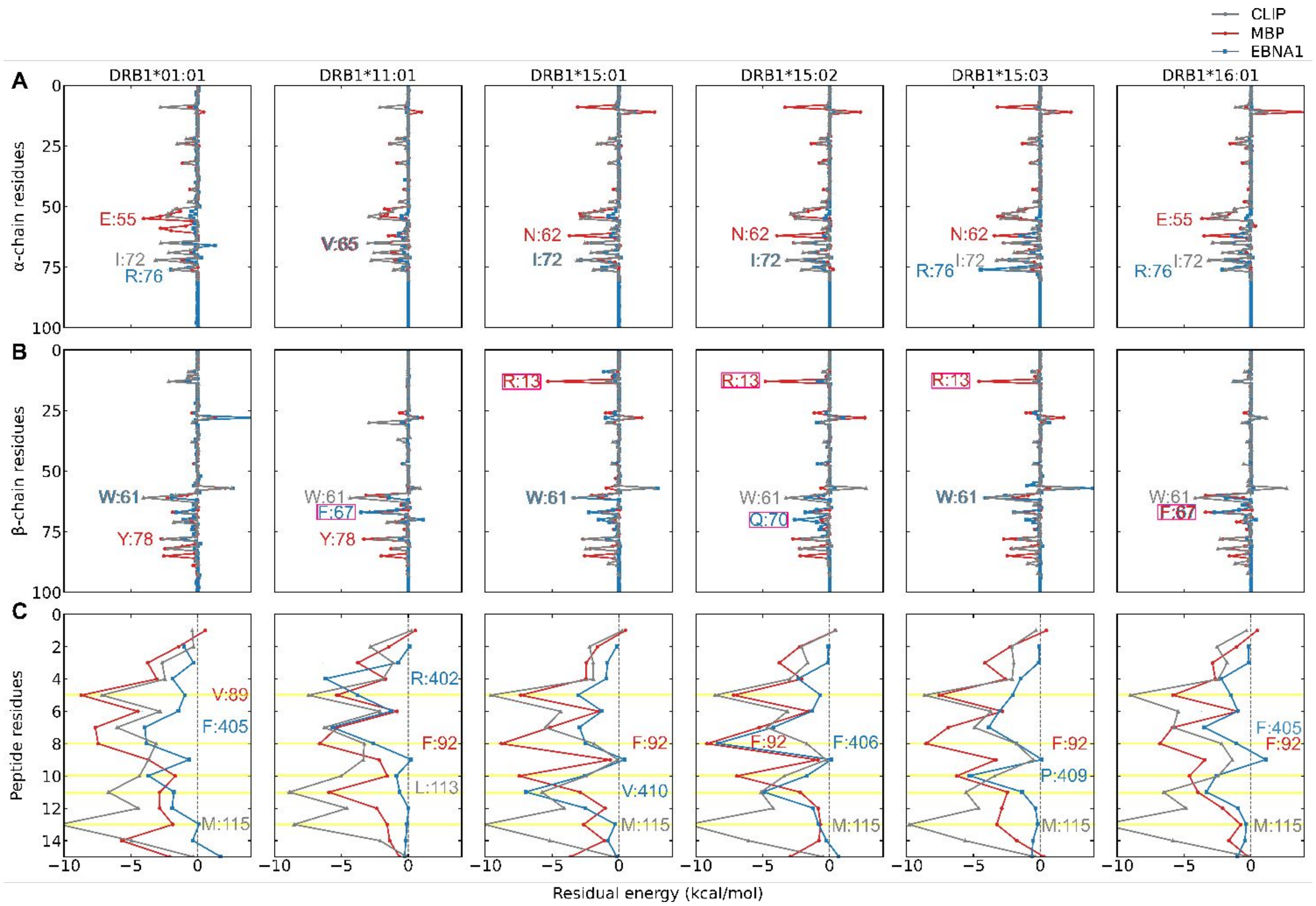

Figure S13. Per-residue binding free energy decomposition obtained by MM/PBSA for HLA-DRB1 complexes associated with the peptides $CLIP_{103\text{-}117}$, $MBP_{85\text{-}99}$, and $EBNA1_{400\text{-}413}$. (A) Per-residue energy contributions along the binding groove region corresponding to the α1 domain of HLA-DRA (α chain). (B) Per-residue contributions along the binding groove region corresponding to the β1 domain of HLA-DRB1 (β chain), with polymorphic positions highlighted by rectangles. (C) Per-residue contributions along the positions of the peptides bound to the groove, shown sequentially (positions 1–15). Regions highlighted in yellow indicate the anchor positions corresponding to pockets P1, P4, P6, P7, and P9 of the binding groove, respectively. The sequences of the analyzed peptides are: $MBP_{85\text{-}99}$ (ENPVVHFFKNIVTPR), $CLIP_{103\text{-}117}$ (PVSKMRMATPLLMQA), and $EBNA1_{400\text{-}413}$ (PGRRPFFHPVGEAD).

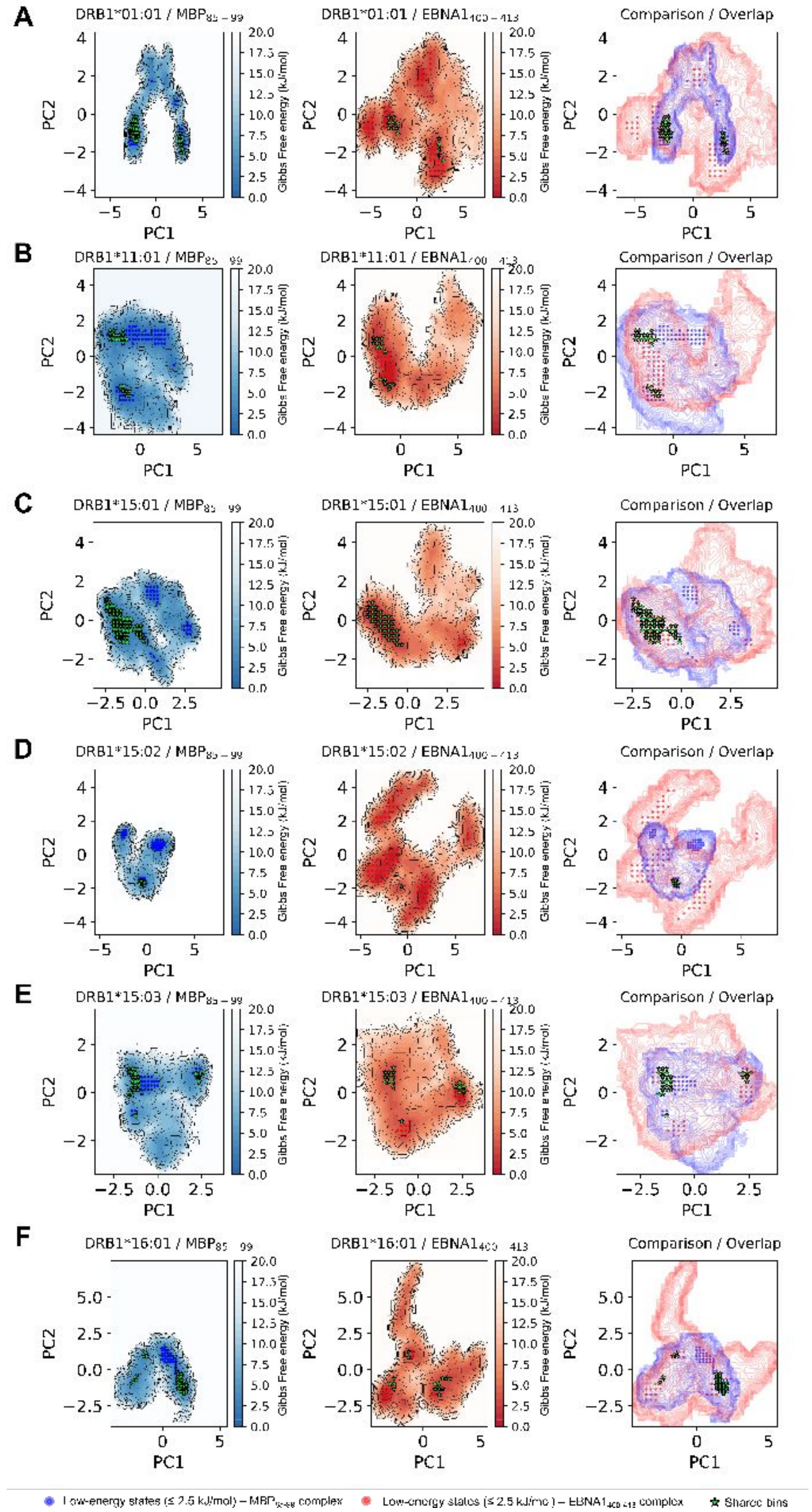

Figure S14. Free energy landscapes (FELs) of the pHLA complexes projected onto PC1 and PC2 for HLA-DRB1 alleles under the $MBP_{85\text{-}99}$ and $EBNA1_{400\text{-}413}$ conditions. For each allele, the individual free energy maps for $MBP_{85\text{-}99}$ (left column) and $EBNA1_{400\text{-}413}$ (center column) are shown, as well as the overlap of low-energy regions (≤ 2.5 kJ/mol) in the PC1–PC2 plane (right column). The FELs were calculated considering the backbone atoms of the binding groove residues and the peptide. Colors represent Gibbs free energy (0–20 kJ/mol), with darker regions indicating more stable states. Panels correspond to the alleles (A) DRB1*01:01, (B) DRB1*11:01, (C) DRB1*15:01, (D) DRB1*15:02, (E) DRB1*15:03, and (F) DRB1*16:01.

**Allele**	MBP low-energy regions	EBNA1 low-energy regions	Frames in shared regions (MBP)	Frames in shared regions (EBNA1)
	n | frames	n | frames	n | frames	n | frames
DRB1*01:01	51 | 22662	65 | 22856	34 | 15732	14 | 5174
DRB1*11:01	80 | 18942	39 | 19814	22 | 5086	15 | 6918
**DRB1*15:01**	**89 | 23405**	**43 | 22873**	**60 | 15449**	**39 | 21289**
DRB1*15:02	49 | 18787	76 | 24847	7 | 2106	1 | 245
**DRB1*15:03**	**54 | 21453**	**34 | 13451**	**27 | 9394**	**18 | 7809**
DRB1*16:01	58 | 20406	28 | 160	28 | 8880	15 | 160

Table S1. Number of low-energy regions (≤ 2.5 kcal/mol) identified in the free energy landscapes (FEL) projected onto the PC1–PC2 space for pHLA complexes containing $MBP_{85–99}$ and $EBNA1_{400–413}$ associated with different HLA-DRB1 alleles. For each peptide condition, the table shows the number of low-energy regions identified and the number of frames belonging to these regions. The table also reports the low-energy regions shared between MBP_{85-99} and $EBNA1_{400-413}$, as well as the number of frames associated with these shared regions for each pHLA complex.

**Model**	**Metric**	**Precision**	**Recall**	**F1-score**	**Support**
$MBP_{85–99}$	Accuracy			0.956 ± 0.005	300006
	Macro avg	0.949 ± 0.003	0.950 ± 0.000	0.950 ± 0.000	300006
	Weighted avg	0.956 ± 0.005	0.956 ± 0.005	0.956 ± 0.005	300006
$EBNA1_{400-413}$	Accuracy			0.993 ± 0.005	300006
	Macro avg	0.998 ± 0.000	0.990 ± 0.000	0.990 ± 0.000	300006
	Weighted avg	1.000 ± 0.000	1.000 ± 0.000	1.000 ± 0.000	300006
$MBP_{85–99}$ + $EBNA1_{400-413}$	Accuracy			0.979 ± 0.003	600012
	Macro avg	0.970 ± 0.000	0.980 ± 0.000	0.972 ± 0.004	600012
	Weighted avg	0.980 ± 0.000	0.979 ± 0.003	0.979 ± 0.003	600012

Table S2. Performance of Random Forest models, evaluated using ten-fold cross-validation, trained on inter-residue distances at the pHLA interface to classify high-risk alleles (DRB1*15:01 and DRB1*15:03) and low-risk alleles (DRB1*01:01, DRB1*11:01, DRB1*15:02, DRB1*16:01).

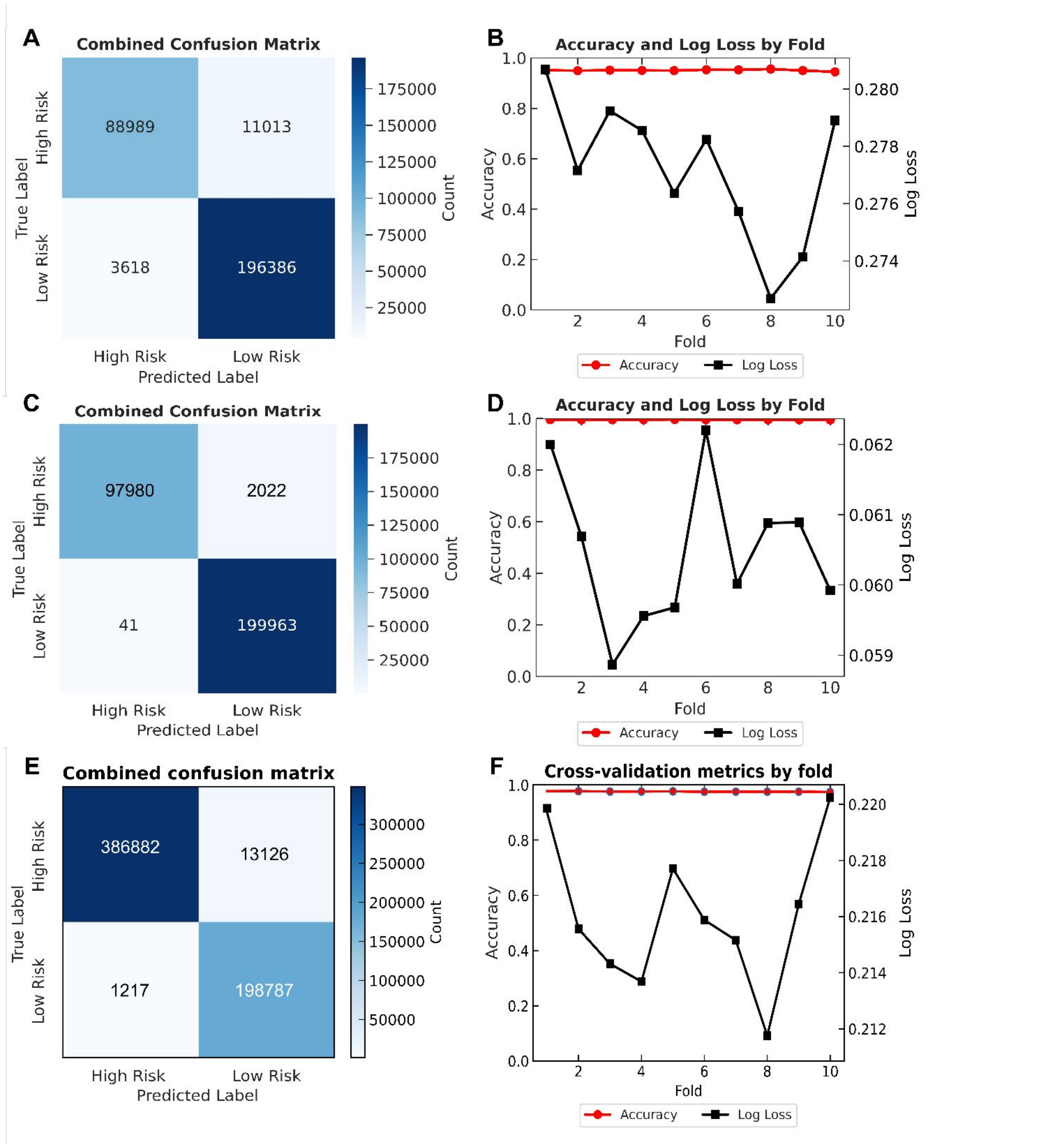

Figure S15. Performance of Random Forest models trained using residue pairs derived from interactions in pHLA complexes. (A–B) model based on MBP85-89; (C–D) model based on EBNA1$_{400-413}$; and (E–F) combined model using MBP$_{85-99}$ + EBNA1$_{400-413}$. Panels A, C, and E show the combined confusion matrices, while B, D, and F, present the cross-validation metrics by fold, including accuracy and log loss.

Model	Class / Metric	Precision	Recall	F1-score	Support
MBP$_{85-99}$	High Risk	0.93	0.93	0.93	100002

Model	Class / Metric	Precision	Recall	F1-score	Support
	Low Risk	0.97	0.97	0.97	200004
	Accuracy			0.96	300006
	Macro avg	0.95	0.95	0.95	300006
	Weighted avg	0.96	0.96	0.96	300006
EBNA1$_{400\text{-}413}$	High Risk	1.00	0.99	0.99	100002
	Low Risk	0.99	1.00	1.00	200004
	Accuracy			0.99	300006
	Macro avg	0.99	0.99	0.99	300006
	Weighted avg	0.99	0.99	0.99	300006
MBP$_{85–99}$ + EBNA1$_{400\text{-}413}$	High Risk	0.97	0.99	0.98	200004
	Low Risk	0.99	0.97	0.98	400008
	Accuracy			0.98	600012
	Macro avg	0.98	0.98	0.98	600012
	Weighted avg	0.98	0.98	0.98	600012

Table S3. Classification performance of the final Random Forest models using pHLA inter-residue distances for discrimination between high-risk and low-risk HLA-DRB1 alleles across different peptide conditions.

Rank	MBP$_{85-99}$			EBNA1$_{400-413}$			MBP$_{85-99}$ and EBNA1$_{400-413}$		
	Pair	CSI ×10⁻³	Norm.	Pair	CSI ×10⁻³	Norm.	Pair	CSI ×10⁻³	Norm.
1	**α:28–C:V88**	**12.457**	**1.00**	**β:57–C:P404**	**22.873**	**1.00**	**β:71–MBP-C:F91 / EBNA1-C:F406**	**14.708**	**1.00**
2	**α:27–C:P87**	**11.423**	**0.92**	**β:54–C:P404**	**20.272**	**0.89**	**β:70–MBP-C:F91 / EBNA1-C:F406**	**13.252**	**0.90**
3	α:26–C:E85	9.625	0.77	β:57–C:H407	17.871	0.78	**β:72–MBP-C:F91 / EBNA1-C:F406**	**12.849**	**0.87**
4	α:26–C:V89	9.346	0.75	β:55–C:H407	15.112	0.66	β:64–MBP-C:N94 / EBNA1-C:V409	11.532	0.78
5	β:65–C:N94	9.272	0.74	β:52–C:P404	14.927	0.65	β:65–MBP-C:K93 / EBNA1-C:P408	10.140	0.69
6	α:27–C:E85	9.109	0.73	β:29–C:R403	14.542	0.64	β:65–MBP-C:N94 / EBNA1-C:V409	8.229	0.56
7	α:26–C:P87	9.001	0.72	β:72–C:P408	14.249	0.62	β:62–MBP-C:K93 / EBNA1-C:P408	7.353	0.50
8	α:26–C:V88	8.851	0.71	β:73–C:P408	14.233	0.62	β:68–MBP-C:K93 / EBNA1-C:P408	6.895	0.47
9	β:18–C:V89	8.705	0.70	β:56–C:H407	13.151	0.70	β:66–MBP-C:K93 / EBNA1-C:P408	6.841	0.47
10	β:64–C:V96	8.255	0.66	β:70–C:V409	13.068	0.57	β:69–MBP-C:F91 / EBNA1-C:F406	6.441	0.44

Table S4. pHLA interface residue pairs with the highest contribution to the classification of HLA-DRB1 alleles associated with lower risk of MS, as identified by machine learning (ML) models. The ten most informative pairs are shown for models trained on complexes containing MBP$_{85-99}$, EBNA1$_{400-413}$, and the combined MBP$_{85-99}$ and EBNA1$_{400-413}$ model. Each pair is represented as a spatial relationship between a residue from the HLA-DRB1 α or β chain and a peptide residue (chain C). For each pair, the class-specific importance (CSI; ×10⁻³) and the corresponding min–max normalized value (Norm.) are reported. In the combined model, equivalent HLA positions are shown together with corresponding residues of the two peptides. Pairs highlighted in bold correspond to those with normalized importance values greater than or equal to 0.8.

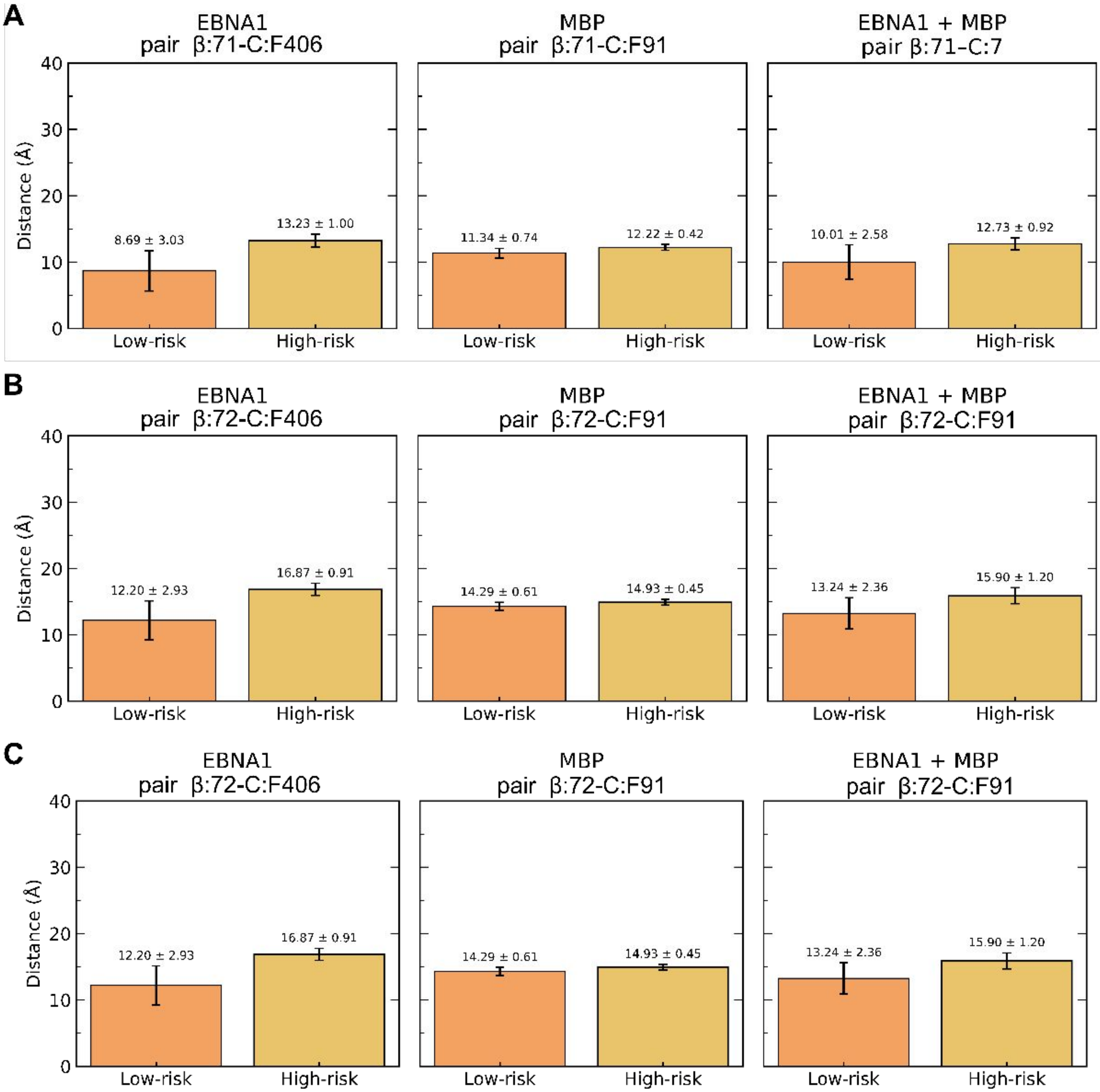

Figure S16. Mean distances of discriminative residue pairs involving position β:71 at the pHLA interface. Bars represent the mean distances (± standard deviation) between residue pairs identified as most informative by the machine learning models, comparing low-risk and high-risk alleles for multiple sclerosis. Equivalent pairs are shown for complexes containing $EBNA1_{400\text{-}413}$ (β:71–C:F406), $MBP_{85–99}$ (β:71–C:F91), and the combined model (β:71–MBP F91/EBNA1 F406). In all cases, higher mean distances are observed in high-risk alleles compared to low-risk alleles. This effect is more pronounced in complexes with $EBNA1_{400\text{-}413}$, whereas differences are more subtle in complexes with $MBP_{85–99}$. The combined model preserves this trend, indicating that position β:71 establishes distinct spatial relationships with equivalent peptide residues, with the magnitude of these differences depending on the peptide condition.

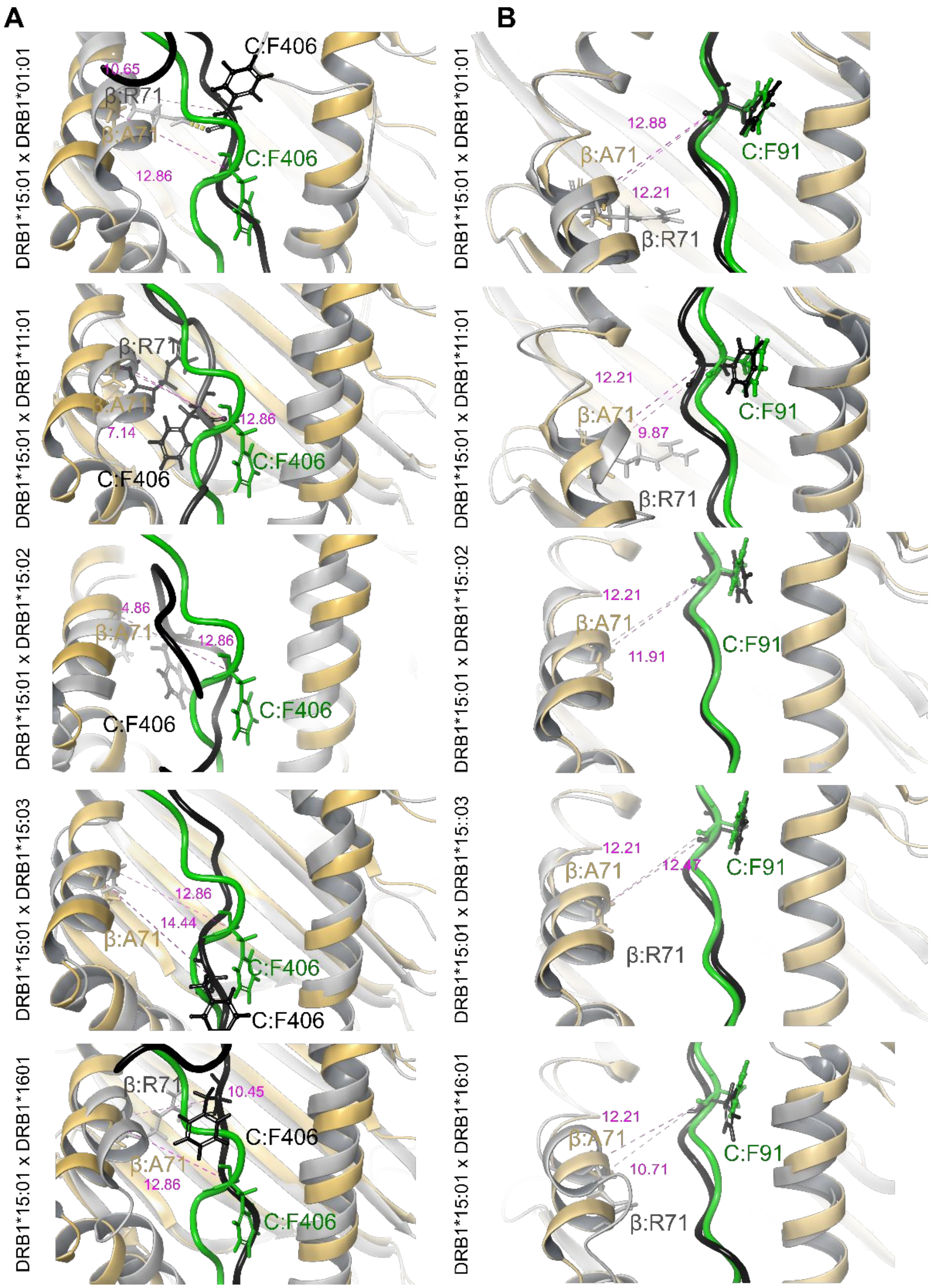

Figure S17. Structural comparison of the dominant frames of pHLA complexes highlighting residue pairs at the interface. (A) Superposition of the dominant frames of complexes containing $EBNA1_{400-413}$, using DRB1*15:01 as the reference, compared with DRB1*01:01, DRB1*11:01, DRB1*15:02, DRB1*15:03, and DRB1*16:01. (B) Equivalent superposition for complexes containing MBP_{85-99}. The reference HLA-DRB1 molecule (DRB1*15:01) is shown in gold, whereas the compared complex is shown in gray. Peptides are shown in green. Distances between selected residues at the pHLA interface are indicated by magenta dashed lines, with values expressed in Å.

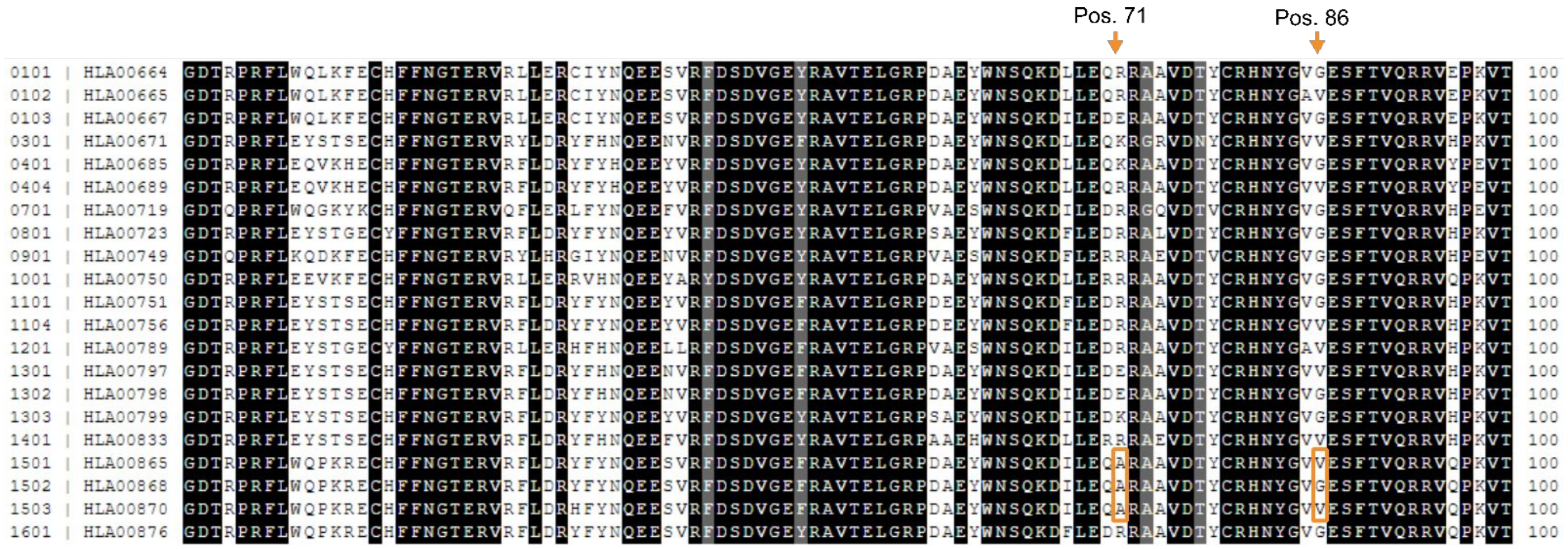

Figura S18. Multiple alignment of different HLA-DRB1 alleles. Areas shaded in black indicate conserved residues, while areas shaded in gray or represented in white indicate regions with similarity or low similarity, respectively.
